# Structure‐based identification of dual ligands at the A_2A_R and PDE10A with anti‐proliferative effects in lung cancer cell‐lines

**DOI:** 10.1186/s13321-021-00492-5

**Published:** 2021-03-03

**Authors:** Leen Kalash, Ian Winfield, Dewi Safitri, Marcel Bermudez, Sabrina Carvalho, Robert Glen, Graham Ladds, Andreas Bender

**Affiliations:** 1 Centre for Molecular Informatics, Department of Chemistry, University of Cambridge, Lensfield Road, CB21EW Cambridge, UK; 2 Department of Pharmacology, University of Cambridge, Tennis Court Road, CB2 1PD Cambridge, UK; 3 Pharmacology and Clinical Pharmacy Research Group, School of Pharmacy, Bandung Institute of Technology, 40132 Bandung, Indonesia; 4 Institute of Pharmacy, Freie Universität Berlin, Königin-Luise-Straße 2 und 4, 14195 Berlin, Germany; 5 Department of Metabolism Digestion and Reproduction, Faculty of Medicine, Imperial College London, SW7 2AZ London, UK; 6 Present Address: GlaxoSmithKline, Gunnels Wood Road, Hertfordshire SG1 2NY Stevenage, UK

**Keywords:** Docking, MD simulations, Structure‐based design, Virtual screening, A_2A_R, PDE10A, Anti‐proliferative, Dual target, Triazoloquinazolines, NSCLC, Lung cancer

## Abstract

Enhanced/prolonged cAMP signalling has been suggested as a suppressor of cancer proliferation. Interestingly, two key modulators that elevate cAMP, the A_2A_ receptor (A_2A_R) and phosphodiesterase 10A (PDE10A), are differentially co-expressed in various types of non-small lung cancer (NSCLC) cell-lines. Thus, finding dual-target compounds, which are simultaneously agonists at the A_2A_R whilst also inhibiting PDE10A, could be a novel anti-proliferative approach. Using ligand- and structure-based modelling combined with MD simulations (which identified Val_84_ displacement as a novel conformational descriptor of A_2A_R activation), a series of known PDE10A inhibitors were shown to dock to the orthosteric site of the A_2A_R. Subsequent in-vitro analysis confirmed that these compounds bind to the A_2A_R and exhibit dual-activity at both the A_2A_R and PDE10A. Furthermore, many of the compounds exhibited promising anti-proliferative effects upon NSCLC cell-lines, which directly correlated with the expression of both PDE10A and the A_2A_R. Thus, we propose a structure-based methodology, which has been validated in in-vitro binding and functional assays, and demonstrated a promising therapeutic value.

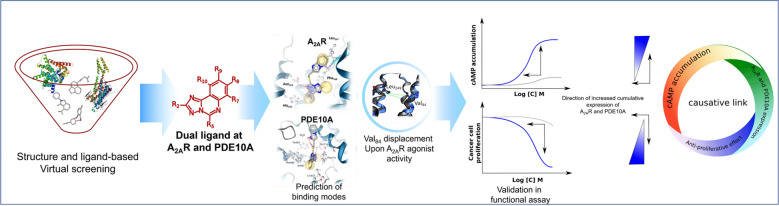

## Introduction

Cyclic adenosine monophosphate (cAMP) is a second messenger that has a major role in transduction and cell signaling in several pathways and biological systems [1]. cAMP elevation may be achieved *via* the activation of the adenylate cyclases by Gs proteins, and the inhibition of cAMP-degrading phosphodiesterases [2], and has been shown to inhibit proliferation of several cancer cell types such as breast cancer, colon cancer, lung cancer, glioblastoma etc [3–6].

Two key modulators of intracellular cAMP are the adenosine A_2A_ receptor (A_2A_R) and the phosphodiesterase 10A (PDE10A), which are often co-expressed in different amounts across NSCLC cell-lines. The A_2A_R is expressed in the two histologically distinct types of NSCLC cell-lines, lung adenocarcinoma and squamous carcinoma cell-lines [7, 8]. Likewise, PDE10A is overexpressed in lung adenocarcinoma, and its inhibition was found to suppress growth [9], demonstrating a correlation between the levels of overexpression and survival [10]. This makes these systems interesting avenues of investigation for relating the amount of co-expression of these two protein targets and their ability to elevate cAMP as well as induce anti-proliferation in these cell-lines.

We hypothesized that a novel approach would be to discover compounds, which act simultaneously as agonists of the A_2A_R that are also inhibitors of PDE10A. cAMP elevation could be achieved through the A_2A_R-Gαs-adenylate cyclase axis, while further promoted by the inhibition of its breakdown *via* PDE10A [7, 8]. A multi-target approach is a departure from standard drug discovery practice, where one target is often the driving force in compound optimization. A multi-target compound could, through synergistic effects, be more effective in elevating cAMP. Indeed, dual PDE inhibition and A_2A_R activation *via* compound combinations exhibited synergy (according to isobologram analysis) in cAMP elevation, and was observed to inhibit proliferation in other cancer cell types such as multiple myeloma and diffuse large B-cell lymphoma [11]. The use of multitarget ligands have also demonstrated beneficial effects on Alzheimer’s and Parkinson’s disease [12, 13]. Therefore, combining this approach in single dual-targeted compounds at the A_2A_R and PDE10A could be explored as a novel anti-proliferative strategy for adenocarcinoma and squamous carcinoma cell-lines.

For the purpose of designing PDE10A inhibitors and A_2A_R agonists, many virtual screening protocols have been reported in the literature, implementing either ligand- or structure-based approaches Examples of ligand-based protocols include *in silico* target prediction, pharmacophore-based and fragment-based approaches and comparative molecular field analysis (CoMFA) [14–19]. Docking, as a structure-based approach, has also been employed for the design of either PDE10A inhibitors or A_2A_R agonists [20]. In addition, molecular dynamics has been used extensively to investigate the conformational dynamics at the A_2A_ adenosine receptor or PDE10A [20–27]. However, none of the reported protocols rationalizes or correctly predicts the functional activity of ligands against the targets of interest, in particular the A_2A_R, which is addressed in this work.

Here, a novel structure-based methodology for identifying ligands that activate the A_2A_R while simultaneously inhibiting the PDE10A is devised. Given that PDE10A is an enzyme, compounds that target the active site would most likely confer inhibition. However, binding to the orthosteric site of the A_2A_R may not guarantee the desired functional activity. For this reason, the structure-based computational approach was focused on the more challenging goal, which involved identifying whether known PDE10A inhibitors are A_2A_R agonists.

The focus of this approach was on the key interacting residues, which are reported in the literature to discriminate between agonist and antagonist activity of A_2A_R ligands [28–31]. It is postulated that the motion of the residue Val_84_ in Transmembrane Helix 3, upon A_2A_R ligand binding, might discriminate between agonist and antagonist activity, which has not previously been studied by any MD approaches [19–24, 32]. Hence, the motion of this residue has been investigated as a conformational descriptor for the characterization of receptor activation by A_2A_R ligands.

Subsequently, the selected compounds were evaluated pharmacologically in vitro using both binding and functional assays. We then extended our studies to evaluate the compounds for their abilities to modulate cell proliferation in lung squamous cell carcinoma and lung adenocarcinoma cell-lines. Their anti-proliferative effects were correlated with the co-expression of the A_2A_R and PDE10A and (increased) cellular levels of cAMP.

## Results

### Method for selecting triazoloquinazolines as candidates for dual ligand activity at A_2A_R and PDE10A

**Fig. 1:**
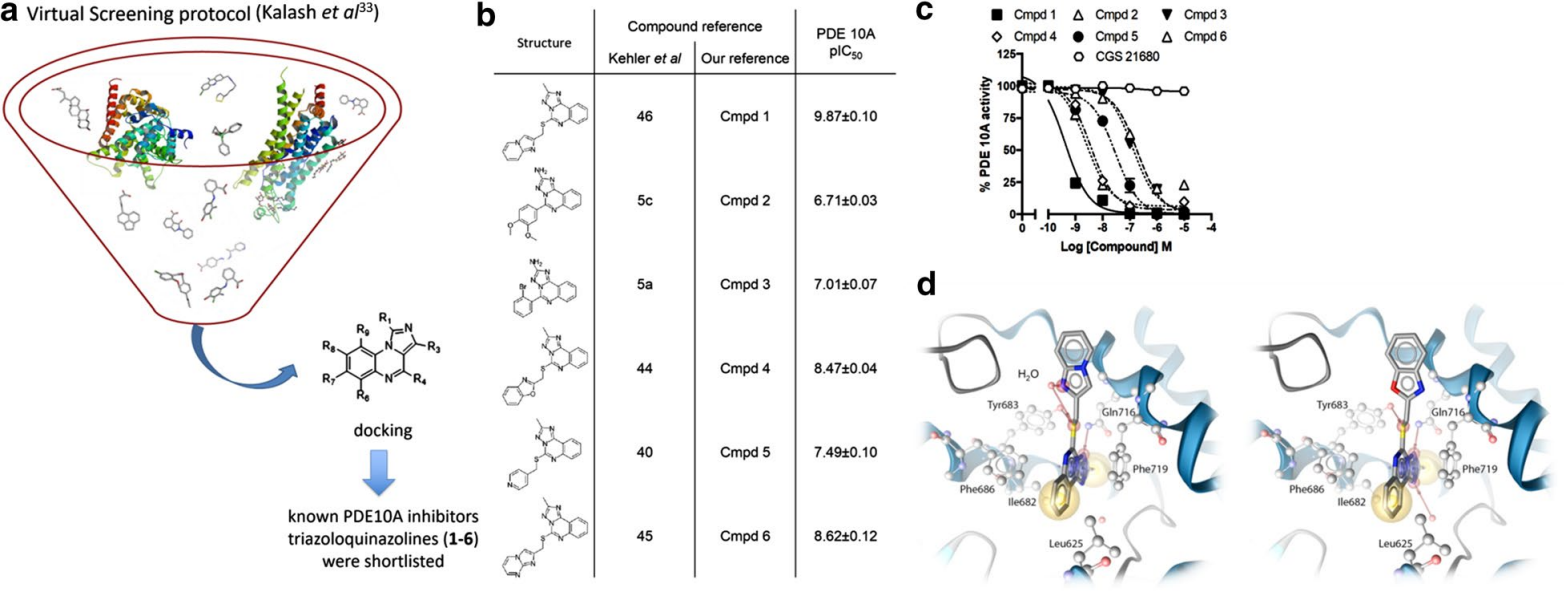
The Structures of the identified PDE10A inhibitors with the potential to bind to the A_2A_R, and their pharmacology at PDE10A. **a** Virtual screening protocol. **b **Chemical structures for the six compounds identified in the *in silico* screen, literature references, compound IDs (used here) and pIC_50_ for PDE10A inhibition. **c **Concentration-response curves generated for **1–6** and CGS21680 at PDE10A. Data is the mean of six individual replicates ± SEM. **d** Representative binding modes proposed for the triazoloquinazolines **1** and **4** docked to the PDE10A crystal structure (PDB ID: 4DDL). Yellow spheres indicate lipophilic contacts. Aromatic interactions are illustrated by purple disks and hydrogen bond acceptors are shown as red arrows. Tyr_683_ is part of the “selectivity pocket” of PDE10A [33], and its interaction through a hydrogen bond with the thioether of both compounds could explain their subtype selectivity

Triazoloquinazolines were identified by Kalash et al. as a compound series that showed the highest frequency of prediction as binders at the A_2A_R and PDE10A by ligand- and structure-based approaches (Fig. 1a) [33]. For the purpose of finding dual-target ligands that elevate cAMP, the focus was on ligands that could simultaneously activate the A_2A_R (agonists) and inhibit PDE10A.

From the ZINC database, six purchasable triazoloquinazolines (**1–6**) were shortlisted (compound **1–6** Fig. 1a, see methods for details) [34], which were (Fig. 1b, c) previously shown to display inhibition of PDE10A (with a rank order of potency of **1** > **6** = **4** > **5** > **3** = **2)** [34]. Importantly, for future reference, no significant activity of the A_2A_R selective agonist CGS21680 at PDE10A was detected. Using a crystal structure of PDE10A (PDB ID: 4DDL) and ligand/protein docking, binding poses were found that appeared consistent (i.e. docking in approximately the same position) for all six compounds (Fig. 1d—illustration of predicted binding modes of representative triazoloquinazolines **1** and **4**). Importantly, this analysis highlighted that the interaction of Tyr683, a residue belonging to a ‘selectivity pocket’ of PDE10A, through a hydrogen bond with the thioether of the compounds could explain their PDE10A subtype selectivity.

Following the initial shortlisting (based on PDE10A activity), compounds **1–6** were docked into the orthosteric site of the A_2A_R protein crystal structure (PDB ID: 2YDO). In this structure a relatively large displacement of the Val_84_ residue was observed (when referenced to its average distance to Leu_249_, a residue that is comparatively static in position relative to the structure as a whole (Additional file 1: Table S1). The relative motion of this amino acid residue is essential for A_2A_R activation, in order to avoid the steric clash that might otherwise result between the agonist and the receptor.

The selection of the structure to be used as the docking model for the A_2A_R was based on the Val_84_-Leu_249_ inter-residue distances found for the active/inactive forms of the A_2A_R protein crystal structures reported in the protein data bank (PDB). Based on this criterion, the A_2A_R crystal structure (PDB ID: 2YDO) was selected since it exhibited the largest inter-residue distance. It was hypothesized that this would allow ligand exploration of a conformational space most likely to be occupied by A_2A_R agonists when docked into the orthosteric site. Indeed, this enabled enrichment of A_2A_R agonists over A_2A_R antagonists and A_2A_R inactives (refer to methods for details). This is in agreement with a previous study by Rodríguez et al. [26], where the A_2A_R crystal structure (PDB ID: 2YDO) displayed the highest enrichment factor value (EF1 %) for docked agonists over the other active crystal structures of the A_2A_R. The 2YDO crystal structure enriched agonists 63.5-fold better than random and 2.9-fold better than antagonists (63.5 % versus 21.9 %) [26]. However, their docking approach failed to find any A_2A_R agonists (which used three active structures: PDB IDs: 2YDO, 2YDV, and 3QAK). The authors rationalized this as resulting from bias of the chosen chemical libraries towards A_2A_R antagonists over agonists.

The evaluation of the six triazoloquinazolines **1**–**6** (Fig. 1a), as promising candidates for A_2A_R agonism, was based on their docking scores. Compounds were selected based on their scores below the score threshold value of -7.33, which was determined as the optimum selection criteria for agonists based on computing the Matthews correlation coefficient (see Methods for more details).

Compounds **1**–**6** were screened against PAINs (PAN Assay Interference Compounds) with regard to the recent analysis of the use of this approach by Tropsha using FAFDrug3 [35], and none of the compounds exhibited any potential PAINs liability.

### Analysis of the molecular docking studies of the representative triazoloquinazolines 1–4 shortlisted for experimental validation

**Fig. 2:**
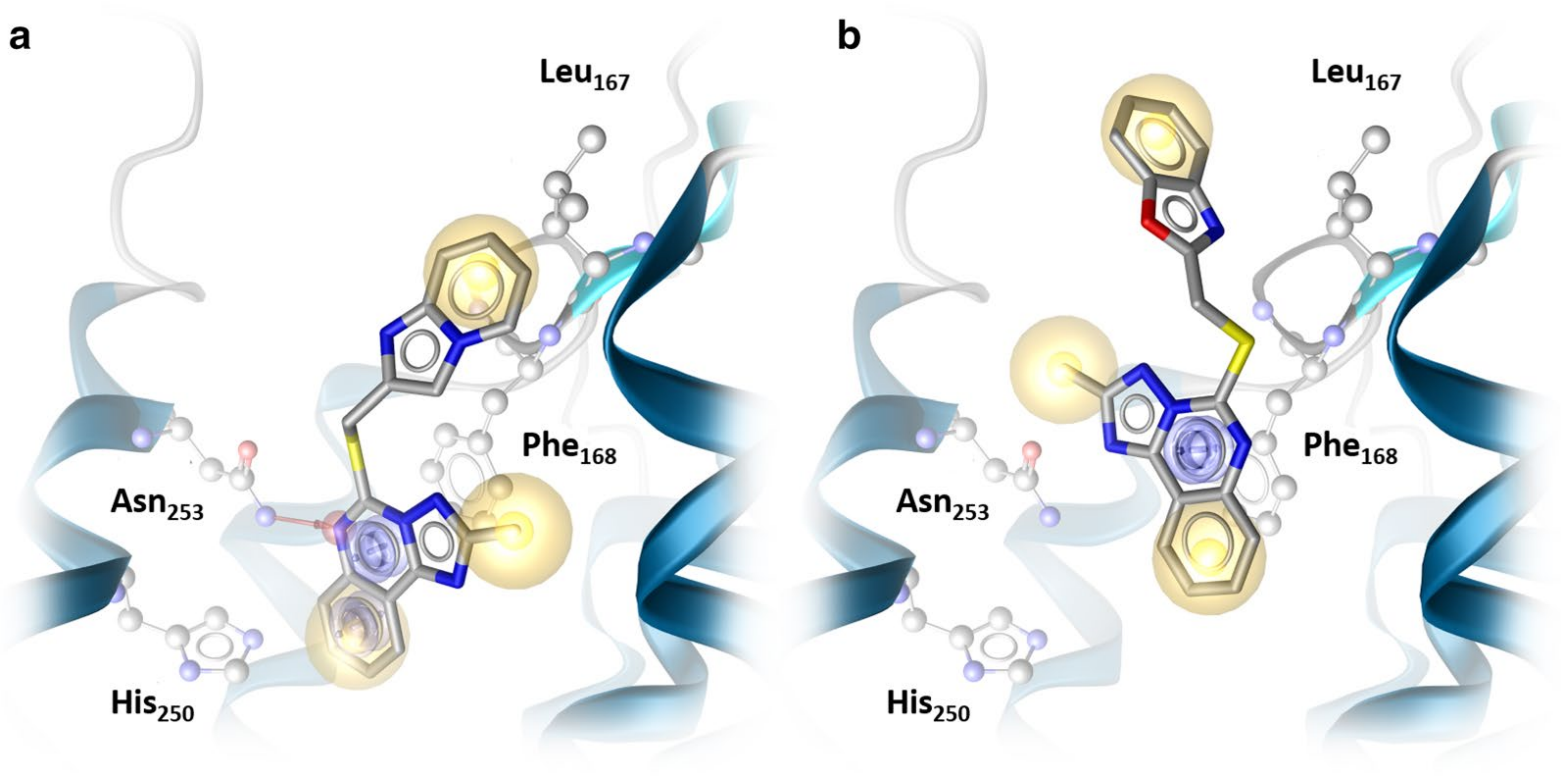
Docking studies predicted molecular interactions similar to those observed for triazoloquinazolines in the A_2A_R protein crystal structure (PDB ID: 2YDO). Distinct binding modes are shown for compounds **1** and **4. a** Compound **1** hydrogen bonds *via* the nitrogen of the quinazoline ring with Asn_253_ and *via* the imidazo ring with Glu_169_. The triazole ring is π-stacked with Phe_168_, and the phenyl group in quinazoline is π-stacked with His_250_ **b** Compound **4** shows π-stacking with Phe_168_. It can be seen that the A_2A_R selective agonist **1** is predicted to bind deeper within the binding site and interacts with His_250_ and Asn_253_, which is consistent with binding modes observed in crystallographic data (PDB IDs: 4UG2, 4UHR, 3QAK, 2YDO and 2YDV). The essential role of His_250_ in shaping the binding site was supported by MD simulation. Yellow spheres indicate lipophilic contacts, red arrows show hydrogen bond acceptors and purple disks indicate aromatic interactions

Docking studies predicted consistent molecular interactions for the triazoloquinazolines, similar to those of the co-crystallized ligand bound to the A_2A_R protein crystal structure (PDB ID: 2YDO). Representative and distinct binding modes are illustrated in Fig. 2. Compounds **1**–**3**, were predicted to be selective A_2A_R ligands, which was attributed to interactions with His_250_ [36, 37]. This residue is located in the core region of the receptor and part of a sub-pocket formed by Leu_85_, Met_177_, Trp_246_ and Leu_249_. Despite the fact that it is conserved among the A_1_R and the A_2A_R subtypes (as suggested by a recent study [38], due to the high conservation of amino acid residues in the adenosine receptor subtypes), subtype selectivity might not be attributed to the receptor-specific amino acid residues, but rather to conformational differences. Also, given that mutation experiments have failed so far to highlight any receptor-specific amino acid residues responsible for subtype selectivity, this would add weight to the suggested hypothesis [37, 39]. Hence, the selectivity of A_2A_R agonists could be attributed to the conformational preferences of the His_250_ amino acid residue that contributes to shaping the orthosteric site to favor their selectivity [38]. Indeed, the interaction with this residue is only observed for the selective A_2A_R co-crystallized agonists, CGS21680 (PDB ID: 4UHR) and UK432097 (PDB ID: 3QAK) but not for the non-selective co-crystallized agonists NECA (PDB ID: 2YDV) and adenosine (PDB ID: 2YDO). These results appear to confirm that interactions with His_250_ serve to improve binding to the lipophilic sub-pocket which suggests this is a driver for A_2A_R sub-type selectivity. In terms of functional activity however, the occurrence of this interaction cannot discriminate between agonists and antagonists [37, 39].

Compound **1** hydrogen bonds *via* the nitrogen of the quinazoline ring with Asn_253_, and *via* the imidazo ring with Glu_169_. The triazole ring is π-stacked with Phe_168_, and the phenyl group in quinazoline is π-stacked with His_250_ (Fig. 2). Compound **4** shows π-stacking with Phe_168_. The selective A_2A_R agonist, compound **1** is predicted to bind deeper within the receptor core and to directly interact with His_250_ and Asn_253_, which is consistent with the experimentally observed interactions between the co-crystallized ligands and the active A_2A_R crystal structures (PDB IDs: 4UG2, 4UHR, 3QAK, 2YDO and 2YDV). The compounds were not predicted to display all the interactions exhibited by the agonist co-crystallized ligands [28–30], in particular the Thr_88_ and Ser_277_ interactions, which are also characteristic of the ZM241385 antagonist [27]. Hence, these interaction types are not characteristic of agonist activity. However, it has been reported in the literature that mutating these residues has a stronger influence on agonist activity than upon the antagonist activity of the A_2A_R ligands, but not on the binding to the A_2A_R [37–39]. As for the co-crystallized A_2A_R antagonists (PDB ID: 5IU4 3UZA, 5K2A, 4EIY, 3EML, 5NM2, 5JTB, 5UVI, and 5UIG), these only show interactions with Phe_168_, Asn_253_, and Glu_169_ residues. Therefore, the type of predicted interaction is not indicative of receptor activation by the triazoloquinazolines. However, the docking model used enriched A_2A_R agonists (exhibited higher docking score distribution) over A_2A_R antagonists and A_2A_R inactives (compounds that do not bind to the A_2A_R). This suggested an investigation (using molecular dynamics) into whether the His_250_ movement would differ between selective versus non-selective A_2A_R agonist binding (discussed in the supporting information) and also to investigate whether the motion of Val_84_ would vary upon agonist and antagonist binding. This could allow discrimination between these different classes of compounds.

### Analysis of MD Simulations reveals that a shift in Val _84_ is one requirement for receptor activation by A_2A_R ligands

**Fig. 3:**
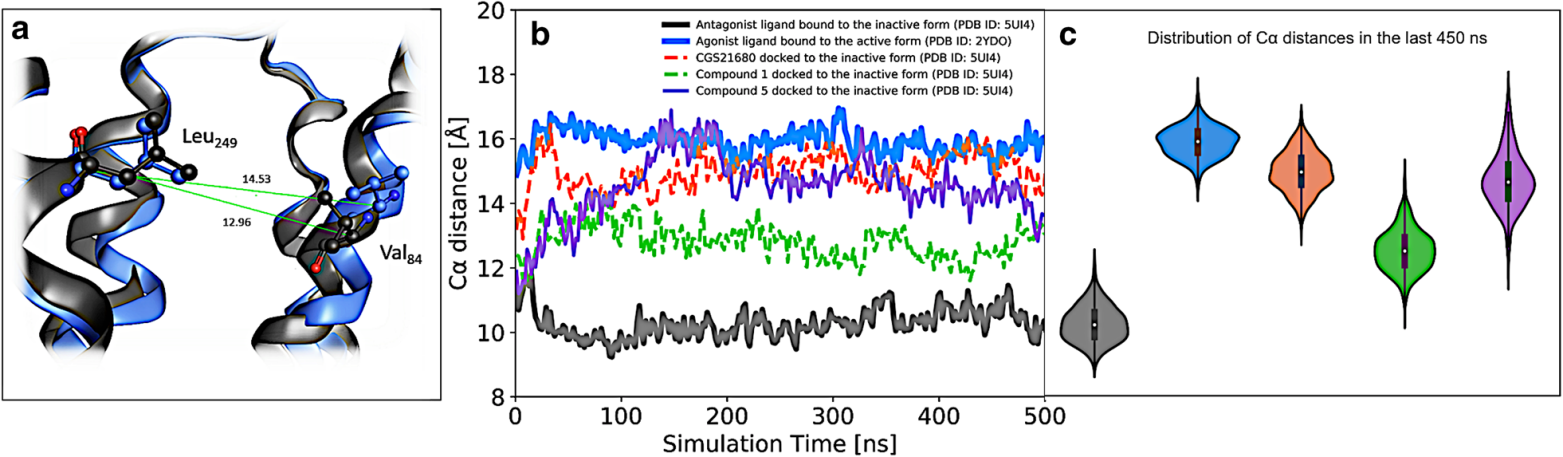
**a** The aligned and superimposed active (PDB ID: 2YDO in blue) and inactive conformations (PDB ID: 5IU4 in grey) of the A_2A_R protein crystal structures. The Val_84_-Leu_249_ Cα distances were measured for the active and inactive conformations and were 14.53 Å and 12.96 Å respectively **b** The moving average trend-lines (bin-size of 20 frames) are for the Val_84_-Leu_249_ Cα distances of the apo structure (PDB ID: 5IU4) and the docked and the co-crystallized structures (PDB ID: 5IU4 and 2YDO use the same color code as 3A) of the A_2A_R over a simulation of 100 ns. Compounds **1**, **5** and CGS21680 are docked into the inactive form of the A_2A_R protein crystal structure (PDB ID: 5IU4). The variations in the computed distances for compounds **1**, **5** and CGS21680 were similar - all increased their average distances over time, moving closer to the average distance observed in the active protein crystal structure (PDB ID: 2YDO). **c** Violin plots for distance distributions (same color code of Fig. 3b) for the last 450 ns of the simulations shows higher Val_84_-Leu_249_ distances for the agonist bound to the A_2A_R in comparison to the antagonist bound to the A_2A_R. Hence, the increase in the Val_84_-Leu_249_ inter-residue distance upon A_2A_R agonist binding serves as a promising conformational descriptor for receptor activation by A_2A_R ligands. A statistical analysis was performed on the distance distributions for the last 450 ns using a Mann-Whitney test and a Kolmogorov-Smirnov test. The differences in medians of the distance distributions for each of the agonists versus the antagonist were significant at a p value < 0.05, and the p value for the Kolmogorov-Smirnov test was < 2.2 × 10^− 16^

The analysis of the active and inactive forms of the available A_2A_R crystal structures is in accordance with reports in the literature, which mention that Val_84_ in TM3 has to move by approximately 2 Å upon agonist binding to avoid a steric clash between the ligand and the receptor [29–31]. This gave rise to the hypothesis that the motion of this residue might discriminate between agonist and antagonist binding (Fig. 3a).

MD simulations (100 ns) were performed for the co-crystallized structures (PDB IDs: 5IU4 and 2YDO), which exhibited the largest differences observed in the distance between the α-carbons of Val_84_ in TM3, and Leu_249_, a relatively fixed residue in TM6 (12.96 Å and 14.53 Å, see methods for details). The same MD analysis was carried out for the apo structure of the A_2A_R (PDB ID: 5IU4), the docked triazoloquinazolines **1**, **4** and **5** with the highest predicted affinities, compound **6** (with lowest predicted affinity), CHEMBL3799351 (a potent antagonist), and CGS21680 (the selective and potent A_2A_R agonist). All these compounds were docked into the orthosteric site of the inactive form of the A_2A_R protein crystal structure (PDB ID: 5IU4), Additional file 1: Figure S4. For the first 50 ns the structures were considered to be relaxing to an annealed state. For the subsequent 50 ns the agonist bound structures showed an increase of the Cα distances between Val_84_ and Leu_249_, with an increased distance equivalent to the active protein crystal structure (PDB ID: 2YDO). Compound **6**, the apo structure, and the antagonist bound structures did not exhibit this increase in Cα distances and instead showed a slight decrease in the Cα distances for the antagonist bound structures in comparison to the apo structure and compound **6**.

To gain further insights from the change in the Cα distances between Val_84_ and Leu_249_ for the agonist bound structures (which are the systems of interest in this study), longer simulations of 500 ns were carried for compounds **1**, **5**, and CGS21680, in addition to the active and inactive cocrystal structures (PDB IDs: 2YDO and 5IU4). The simulations were run in duplicate.

The same trends were observed in the longer simulations. Over the first 50 ns the structures were annealing, and for the rest of the simulation (the last 450 ns) compounds **1**, **5** and CGS21680 increased their Cα distances between Val_84_ and approaching the distance observed for the active protein crystal structure (PDB ID: 2YDO), as shown in Fig. 3b, c. Hence, the increase in the distance between Val_84_ and Leu_249_ residues observed upon A_2A_R agonist binding appears to serve as a useful conformational descriptor for receptor activation by A_2A_R ligands.

### Characterisation of triazoloquinazolines affinity constant at adenosine A_2A_R

**Fig. 4:**
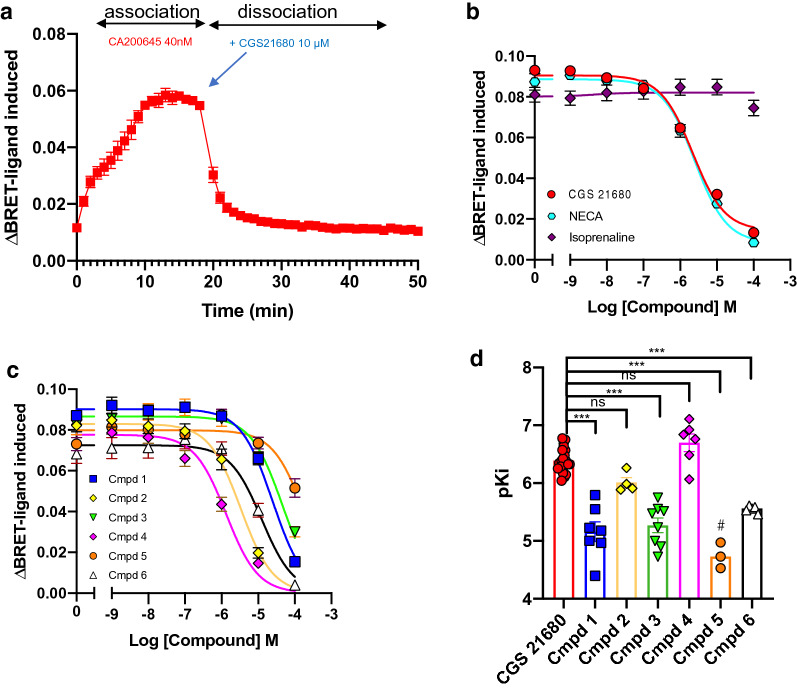
Characterisation of ligands targeting A_2A_R/PDE10A using a NanoBRET-based ligand binding assay. **a** Kinetic binding curve of CA200645 at Nluc-A_2A_R expressed HEK293T cells. After 19 minutes association with 40 nM CA200645, CGS21680 was injected to give a final concentration of 10 µM in order to displace the fluorescent probe. The curve was fit into “association then dissociation” model built in Prism 8.4.3. **b** Competition of CA200645 (300 nM) by reference compounds including CGS21680, NECA, and isoprenaline at equilibrium. **c** Competitive binding curves of triazoloquinazolines in correspond to of 300 nM CA200645. Both curves (panel B and C) were fitted using the “one-site Ki” equation where K_D_ and concentration of hot ligand were set to 65 nM and 300 nM, respectively. Data points are the mean ± SEM from 3–27 repeats performed in duplicate. (D) The summary of binding affinities (pK_i_) of tested ligands. pK_i_ values were calculated from inhibition of CA200645 binding at equilibrium to Nluc-A_2A_R-expressed HEK293T cells. ^#^ Cmpd 5 did not fully displace binding of CA200645 under condition tested. Statistical significance (*p < 0.05; **p < 0.01; ***p < 0.001; ****p < 0.0001) compared to CGS21680 was determined by one-way ANOVA with *Dunnett’s post-test*

We sought to validate the docking studies by quantifying the affinity of each compound at the A_2A_R using a NanoBRET binding assay. In this experiment, we used N-terminally tagged A_2A_R with Nanoluciferase (Nluc) that will emit bioluminescence in close proximity with the fluorescent probe, CA200645, in the presence of Nluc substrate. Firstly, we determine the affinity constant of CA200645 in our expression system. CA200645 has been extensively used to characterise ligand binding properties at adenosine receptor subtypes [40–42]. Using HEK293 cells we determined the disassociation constant (K_D_) of CA200645 at the Nluc-A_2A_R to be 65 nM (Fig. 4a). We next extended our studies to use a classical competition binding assay ([43, 44]) where non-fluorescent ligands compete for binding at the Nluc-A_2A_R with CA200645. Using this approach, we determined the pKi for NECA as 6.36 ± 0.09 and CGS21680 as 6.39 ± 0.04 while isoprenaline (a non-selective agonist of β-adrenoceptors) failed to displace CA200645 (Fig. 4b). We next determined the rank order of affinities for the six triazoloquinazolines compounds at the A_2A_R to be: cmpd **4** > cmpd **2** > cmpd **6** > cmpd **1** = cmpd **3** > cmpd **5** (note: under condition tested, cmpd **5** was unable to fully displace CA200645) (Fig. 4c, d).

### Identifying AR subtype selectivity of triazoloquinazolines

**Fig. 5:**
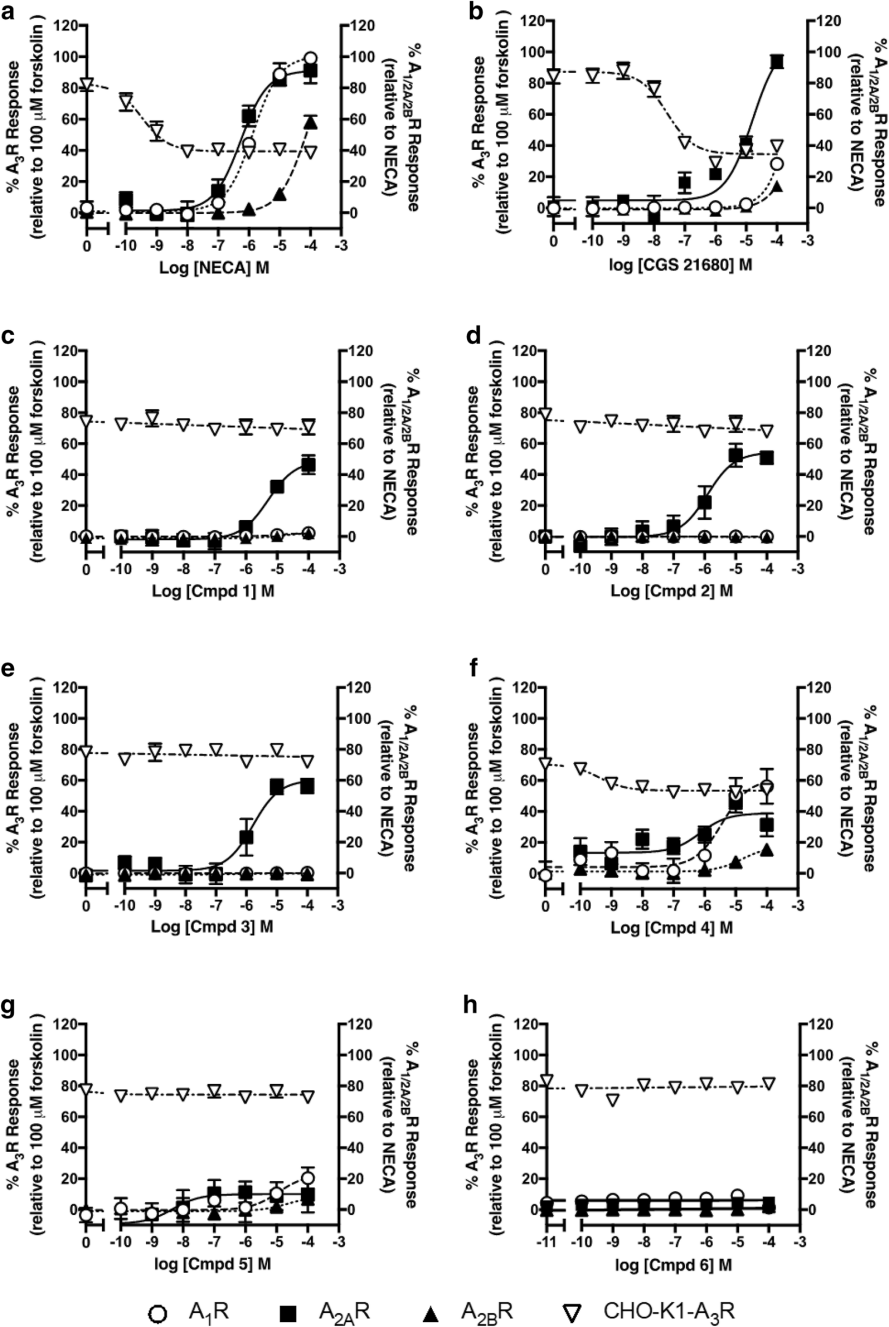
Dose-response curves for NECA, CGS21680 and compounds **1**–**6** in either the A_1_R and GPA1/Gα_i1/2_, A_2A_R and GPA1/ Gα_s_, or the A_2B_R (with GPA1/Gα_s_ expressed in yeast strains). The efficacy of the compounds (**1**–**6**) was measured against A_3_R in CHO-K1-A_3_R cells. Reporter gene activity in yeast was determined using β-galactosidase assays, after 16-hours stimulation with either: NECA (**a**), CGS21680 (**b**) compound **1** (**c**), compound **2** (**d**), compound **3** (**e**), compound **4** (**f**), compound **5** (**g**), compound **6** (**h**), whereas cAMP inhibition was determined when in CHO-K1-A_3_R cells which were co-stimulated with each of the compounds **1**–**6** and 1 µM Forskolin. In general, the triazoloquinazolines **1**–**5** exhibited agonistic activity against the adenosine receptor sub-types, with compounds **1**–**3** being selective A_2A_R agonists. The data is represented as either percentage of the response obtained upon stimulating each receptor (A_1_R, A_2A_R, or A_2B_R) with NECA stimulation, or as a percentage response relative to 100 µM Forskolin simulation in the A_3_R ± SEM of 4–6 individual replicates

Identification of AR subtype selectivity of triazoloquinazolines was performed using previously characterised yeast strains expressing human A_1_R, A_2A_R or A_2B_R [45]. The A_3_R cannot be functionally expressed in yeast (Knight et al., 2016), therefore we utilised CHO-K1 cells stably expressing A_3_R (CHO-K1-A_3_R). Testing the compounds in these systems identified compounds **1**, **2**, **3**, **4**, **5** to be A_2A_R agonists, whilst compounds **1**, **2**, **3** are A_2A_R-selective (Fig. 5, Additional file 1: Table S2). It is interesting to note that compound **6** was able to bind to the A_2A_R but given that in the yeast based assay it failed to elicit a functional response, we suggest it maybe an A_2A_R antagonist.

**Fig. 6:**
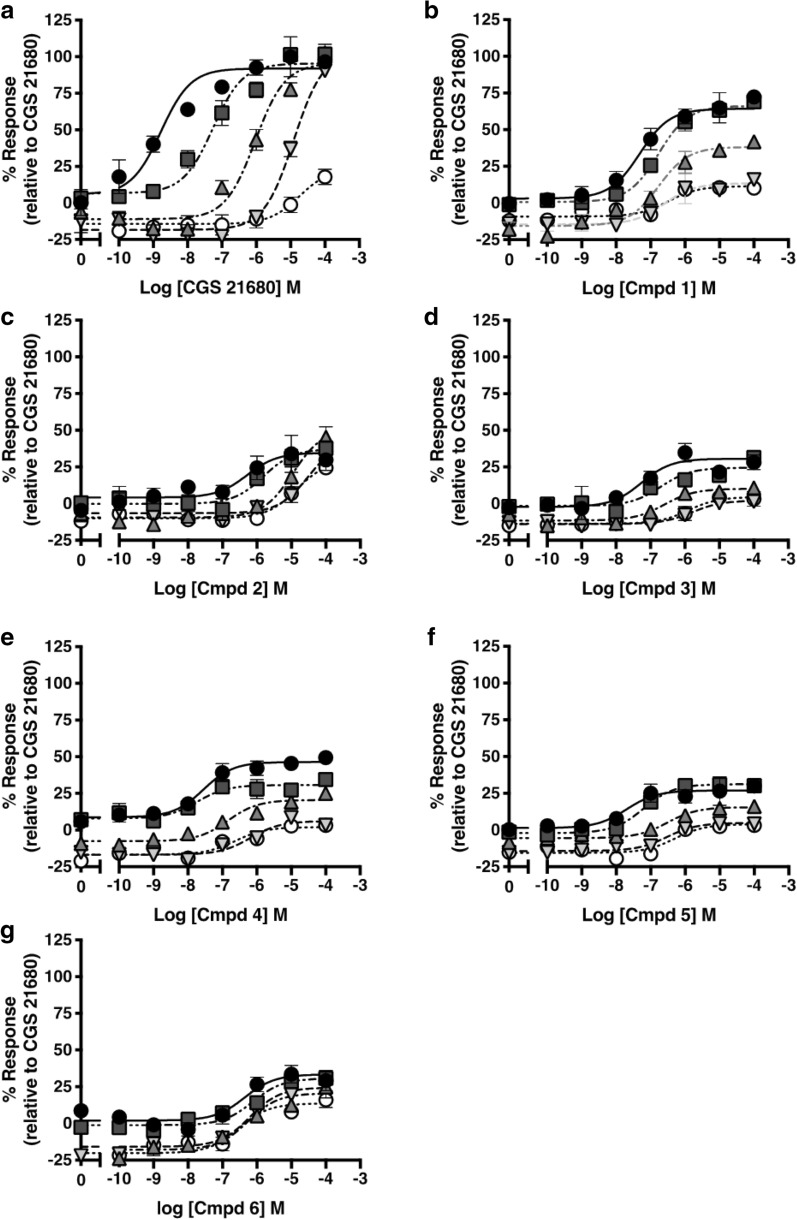
CGS21680 and compounds **1**–**6** elevated cAMP in A_2A_R stably expressed in CHO-K1 cells, which were antagonized by ZM241385. A_2A_R stably expressed in CHO-K1 cells (CHO-K1-A_2A_R) were stimulated for 30 minutes with: CGS21680 (**a**), compound **1** (**b**), compound **2** (**c**), compound **3** (**d**), compound **4** (**e**), compound **5** (**f**), or compound **6** (**g**), after which the cAMP levels were determined. Subsequently compounds were antagonized with either 100 pM, 10 nM or 1 µM ZM241385, which decreased the cAMP levels to the same level of CHO-K1 cells (no A_2A_R stably expressed). Data represented are relative to the response of CGS21680, ± SEM of 4–9 individual replicates

To further verify the efficacy of compounds against the A_2A_R, we assayed their ability to stimulate cAMP production using CHO-K1 cells stably expressing human A_2A_R (CHO-K1-A_2A_R). All compounds tested were observed to be partial agonists, relative to CGS21680, with a rank order of potency of CGS21680 > **5** = **4** > **1** = **3** > **6** > **2** (Fig. 6; Table 1). Antagonism with ZM241385 displayed non-classical antagonism, which is presumed to be due to the dual effects upon endogenous PDE10A (Additional file 1: Figure S5). For compound **6**, treatment with ZM241385 solely reduced E_max_ and basal levels, with no effect on the response range (Fig. 6; Table 1). ZM241385 has been suggested to be an inverse agonist at the A_2A_R potentially explaining these effects [30]. Importantly, all compounds were able to stimulate cAMP production in the absence of the A_2A_R, or in the presence of 1 µM ZM241385, presumably from inhibition of PDE10A. Thus, we observe a significant increase in efficacy of compounds **1–5** via the additional action upon the A_2A_R (Fig. 6; Table 1), which could be attributed to an additive action in elevating intracellular cAMP levels.

**Table 1:** Potency (pEC_50_) and range of responses for cAMP production upon CGS21680 and triazoloquinazoline stimulated cAMP accumulation in CHO-K1-A_2A_R and CHO-K1 cells

	CHO-K1-A_2A_R			CHO-K1			CHO-K1-A_2A_R vs. CHO-K1	
	pEC_**50**_^**a**^	Range^**b**^	n	pEC_**50**_^**a**^	Range^**b**^	n	Δ pEC_**50**_^**c**^	Δ Range^**d**^
CGS21680	8.78 ± 0.2	86.33 ± 7.2	9	ND	ND	4	–	–
Cmpd 1	7.32 ± 0.2	61.14 ± 5.2^***^	8	6.49 ± 0.3	20.19 ± 2.7	4	0.83 ± 0.5	40.95 ± 7.80
Cmpd 2	6.29 ± 0.5^**^	30.50 ± 8.1	6	4.85 ± 0.2	39.46 ± 3.9	4	1.44 ± 0.6	− 8.96 ± 10.6
Cmpd 3	7.26 ± 0.3^**^	28.95 ± 6.3	6	5.90 ± 0.3	18.32 ± 2.3	4	1.21 ± 0.5	10.63 ± 8.70
Cmpd 4	7.55 ± 0.2	37.71 ± 2.9^**^	5	6.62 ± 0.2	18.75 ± 1.7	4	0.93 ± 0.6	18.96 ± 3.60
Cmpd 5	7.70 ± 0.4^**^	27.42 ± 4.4	6	6.30 ± 0.2	19.49 ± 1.7	4	1.28 ± 2.4	7.93 ± 6.1
Cmpd 6	6.52 ± 0.4	33.87 ± 5.3	6	6.35 ± 0.3	31.42 ± 4.6	4	0.17 ± 0.8	2.45 ± 9.9

Data ± SEM of *n* individual replicates. ^a^Negative logarithm of agonist concentration producing half-maximal response. ^b^Percentage range of response observed upon agonist stimulation, relative to that obtained with CGS21680 stimulation in each cell type. ^c^Change in pEC_50_ between CHO-K1 and CHO-K1-A_2A_R cells (Δ pEC_50_ = pEC_50_(CHO-K1-A_2A_R) - pEC_50_(CHO-K1)). ^d^Change in range between CHO-K1 and CHO-K1-A_2A_R cells (Δ Range = Range (CHO-K1-A_2A_R) - Range (CHO-K1)). *ND* Not determined, full dose-response curve not feasible. Statistical difference, between CHO-K1-A_2A_R cells and CHO-K1 cells, was calculated using pair-wise t-tests, for each agonist (*p < 0.05, **p < 0.01, ***p < 0.001)

### Dual PDE 10A inhibition and A_2A_R agonism is anti‐proliferative in CHO-K1-A_2A_R cells

Both CHO-K1 and CHO-K1-A_2A_R cells displayed concentration-dependent inhibition of cell proliferation when stimulated with forskolin (Additional file 1: Figure S6, Table S3), confirming the anti-proliferative effects of cAMP. However, sole activation of the A_2A_R, via CGS21680 stimulation, had no anti-proliferative effects upon either cell type (Additional file 1: Figure S6, Table S3). In contrast stimulation with compound **1** displayed A_2A_R-dependent inhibition of cell growth. Compounds **3–5** show anti-proliferative effects in CHO-K1 cells, which increased in terms of both potency and efficacy when the A_2A_R was expressed (Additional file 1: Figure S6, Table S3). Compound **2** appeared to be anti-proliferative regardless of the cell type tested whereas Compound **6** displayed little anti-proliferative action implying that that sole inhibition of PDE 10A has little effect upon the proliferation of CHO-K1 cells (Additional file 1: Figure S6, Table S3).

### Dual PDE 10A inhibition and A _2A_ R agonism is anti-proliferative in Lung carcinoma cells

**Fig. 7:**
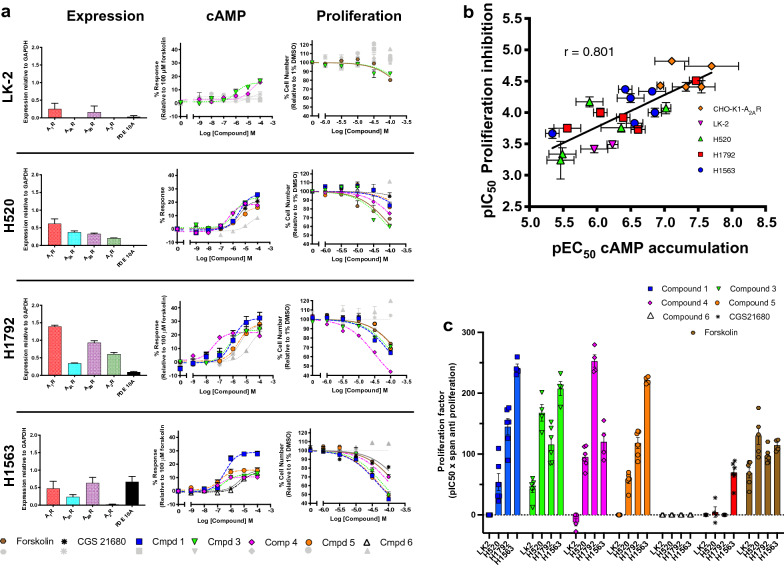
Lung squamous cell carcinoma and adenocarcinoma cells display increasing sensitivity to compounds **1**, **3–5** in terms of proliferation, dependent upon combined A_2A_R and PDE10A expression. Lung squamous cell carcinoma cells (LK-2 and H520) and lung adenocarcinoma cells (H1792 and H1563 were subjected to semi-quantitative RT-PCR analysis to determine expression of the A_1_R, A_2A_R, A_2B_R, A_3_R and PDE 10A, data represented relative to GAPDH expression, ± SEM of 3 individual replicates. Further, each cell line was stimulated with CGS21680 or compounds **1**, **3–6** for 30 minutes and cAMP levels determined. Data represented relative to the response obtained upon stimulation with 100 µM Forskolin, ± SEM of 4–8 individual replicates. Additionally, all cells were stimulated with CGS21680, or **1**, **3–6** for 72 hours and cell number determined using CCK-8. Data represented as a percentage of the cell number present after treatment with 1 % DMSO, ± SEM of 4–8 replicates. (B) Correlation plot for pEC_50_ of each compounds ability to stimulate cAMP production vs. its pIC_50_ for inhibiting proliferation. Data represented ± SEM. (C) Proliferation factor (pIC50 x span anti-proliferative  Additional file 1: Table S5) calculated for **1**, **3–6**, CGS21680s and forskolin at LK-2, H520, H1792 and H1563 cells. Bars represent the mean I_max_ ± SEM, whilst individual data points are shown as a scatter plot.

Having established that the compounds **1–5** appear to have dual activity in CHO-KI cells where the A_2A_R was over expressed we then extended our studies to a series of lung carcinoma cells: two lung squamous cell carcinomas (LUSC): LK-2 and H520, and two lung adenocarcinoma cells (LUAC): H1563 and H1792, which express differing levels of the four adenosine receptor subtypes and PDE10A (Fig. 7a). Using these cell lines, we investigated the effects of compounds of our dual-target compounds upon cAMP production and proliferation (Fig. 7). Note, compound **2** was not analysed for cAMP production in this study due to apparent off-target toxic effects upon CHO-K1 cell proliferation—a feature also noted in all four lung carcinoma cell lines.

LK-2 cells express the A_1_R, A_2B_R and very low levels of PDE10A, but lacked expression of the A_2A_R (Fig. 7a). In these cells compound **3** and to a lesser extent compound **4** were able to stimulate cAMP production (Fig. 7a, Additional file 1: Table S4). However, only forskolin and compound **3** (Fig. 7a, Additional file 1: Table S5) displayed any anti-proliferative actions. Thus, in the absence of significant PDE10A or A_2A_R expression, compound **1** and **5** displayed little activity. Compound **4** is an agonist for the A_2B_R so presumably this explains its ability to stimulate cAMP production. The action of compound **3** was somewhat of a surprise and may suggest it has additional activities beyond A_2A_R and PDE10A. By means of a comparison, H520 cells express all four ARs, but no PDE 10A. In these cells, we were able to observe stimulation of cAMP accumulation when exposed to all compounds except for compound **6**, which displayed low potency and efficacy (Fig. 7, Additional file 1: Table S4). The increase in activity of the compounds was also apparent for proliferation assays, where compounds **1**, **3**–**5** all displayed anti-proliferative activity with higher affinity and efficacy than that observed in the LK-2 cells (Fig. 7, Additional file 1: Table S5). This data highlights the potential of the compounds to prevent proliferation when the A_2A_R is expressed. Likewise, in H1792 cells we observe the expression of all four ARs and an increase in PDE10A expression, relative to H520 cells (Fig. 7). Again, we observed the ability of all compounds to elevate cAMP levels, whilst compounds **1**, **3, 4** and **5** act in an anti-proliferative manner (Fig. 7, Additional file 1: Tables S4, S5). The same was also apparent for H1563 cells, which in contrast to H1792 cells express much higher levels of PDE10A (Fig. 7, Additional file 1: Tables S4, S5). By comparing the observed potencies for proliferation and cAMP assays, across all cell types, for all anti-proliferative compounds, a strong correlation was observed (Fig. 7B, r = 0.80, 95 % CI; 0.85–0.91). This suggests that through improving efficacy in terms of cAMP production, an increased efficacy can also be achieved in terms of proliferation inhibition.

Finally, to provide convenient means by which to compare the anti-proliferative activities of the compounds tested in this study, we multiplied the potency term (affinity) for the compounds by their efficacy (span of antiproliferation)—generating a ‘proliferation factor’ term as described previously [46]. Using this analysis, we can observe that compounds **1**, **3, 4** and **5**, all display improved efficacy when both PDE10A and A_2A_R are present in the cells (Fig. 7). In contrast, compound **6** displays no anti-proliferative activity in any cell type tested whilst CGS21680 is only anti-proliferative in H1563 cells (Fig. 7, Additional file 1: Table S5), suggesting these are more sensitive to proliferation inhibition. In contrast, forskolin displays near equal activity in all cell types tested. As described earlier, compound **3** displayed activity in all four NSCLC cell lines suggesting it may display off target effects. Significantly, it is worth highlighting that compound **4** displayed higher efficacy when the A_2B_R was most abundantly expressed in cells. This directly corelates with it being non-selective at the different AR subtypes and suggests it may be a pan-AR/PDE10A compound.

## Conclusions

In this work, a novel structure-based approach has been successful in identifying triazoloquinazolines as the first dual ligands that activate the A_2A_R and inhibit PDE10A simultaneously. Docking of the triazoloquinazolines **1**–**6**, which are known PDE10A inhibitors, was performed on the orthosteric site of the A_2A_R (PDB ID: 2YDO). It is demonstrated experimentally using a BRET-based ligand-binding assay that these ligands indeed bind to the A_2A_R. The rank order of affinity for the six triazoloquinazolines at the A_2A_R was found to be: cmpd **4** > cmpd **2** > cmpd **6** > cmpd **1** = cmpd **3** > cmpd **5**.

Functional analysis in yeast-screening assay and in mammalian cells demonstrated that compounds **1**–**5** were A_2A_R agonists and revealed that compounds **1**–**3** are selective for the A_2A_R. It is suggested that the observed A_2A_R sub-type selectivity for **1**–**3** is attributed to their predicted interactions with the His_250_ residue, which is an interaction present only in the selective co-crystallized A_2A_R agonists, such as CGS21680 and UK432097. It was further demonstrated by MD simulation analysis that this residue undergoes conformational changes only when selective A_2A_R agonists are bound and not when non-selective agonists bind to A_2A_R. This could contribute to shaping the orthosteric site to favor selectivity of A_2A_R agonists. Moreover, MD analysis highlighted the motion of Val_84_ in TM3 as an essential requirement for A_2A_R activation.

Compounds **1** and **3**–**5** exhibited promising concentration-dependent anti-proliferative effects in lung squamous cell carcinoma cells and lung adenocarcinoma cells, which correlated with co-expression of A_2A_R and PDE10A and increased cellular levels of cAMP. Compound **1** (as a selective A_2A_R agonist and a PDE10A inhibitor) exhibited increased potency for both cAMP accumulation and anti-proliferative actions, which increased in tandem with the combined target expression (A_2A_R and PDE10A) across the NSCLC cell lines, from LK-2-H520-H1792-H1563. Hence, the structure-based approach proposed in this work has been successfully validated using binding and functional assays, and it provides a template for generating A_2A_R agonists as part of a dual-target design objective.

## Methods

### Design approach for the discovery of dual ligands at the A_2A_R and PDE10A

Triazoloquinazolines were shortlisted as candidates of dual ligands at the A_2A_R and PDE10A since this chemical series were predicted to show activity based on ligand- and structure- based techniques [33]. The focus was on discovering compounds that elicited an elevation of cAMP by the activity of ligands having dual effects, simultaneously agonists at A_2A_R and inhibitors of PDE10A.

From the ZINC database, eleven purchasable triazoloquinazolines that were experimentally determined as PDE10A inhibitors were identified using a search for the triazoloquinazoline substructure with the following criteria: Uniprot ID: Q9Y233 and IC_50_ < 10 µM. Identified triazoloquinazolines had the following ZINC IDs: 3,154,141, 3,141,002, 6,206,233, 9,937,921, 9,939,949, 2,968,902, 14,728,559, 424,907, 13,229,753, 44,924,158, and 8,747,709. These were downloaded for subsequent docking into the orthosteric site of the A_2A_R protein crystal structure.

### Selection of the A_2A_R protein crystal structure for shortlisting triazoloquinazoline candidates as A_2A_R agonists

All the active forms of the A_2A_R protein crystal structure with the following PDB IDs (4UG2, 4UHR, 3QAK, 2YDO, and 2YDV) and the inactive forms with the following PDB IDs (5IU4, 3UZA, 5K2A, 4EIY, 3EML, 5NM2, 5JTB, 5UVI, and 5UIG) were downloaded into MOE [47]. It has been reported in the literature that Val_84_ in TM3, which is located in the orthosteric site, has to shift its position upon agonist binding owing to a steric clash with the ligand, which may contribute to the 2 Å shift observed in H3 [29–31]. To evaluate the change in the interaction upon agonist binding, the distance was calculated from a single amino acid residue to Val_84_. This gave a frame of reference to compare structures. The ‘fixed’ amino acid residue selected was Leu_249_ in TM6. This was achieved by aligning all the active and inactive forms of the A_2A_R protein crystal structures (using the sequence editor > alignment > align/superimpose option). Then, the mean RMSD displacement from the mean of all the aligned structures was calculated for Leu_249_, which turned out to be low (0.40 Å) confirming that it is reasonably static in its relative position.

For each PDB ID of the active and inactive forms of the A_2A_R crystal structures, the distance between the α-carbons of Val_84_ in TM3 and Leu_249_ in TM6 was measured in MOE using the measure > distances option. Additional file 1: Table S1 lists all the Val_84_-Leu_249_ inter-residue distance values. The inter-residue distances of the active forms ranged from 14.30 to 14.53 Å, and for the inactive forms they ranged from 12.96 to 13.36 Å. The largest displacement of the Val_84_ residue was measured for the active form in PDB ID: 2YDO, and the distance was equal to 14.53 Å. This can be compared to the inactive form of the A_2A_R protein crystal structure (PDB ID: 5IU4), which had the minimum distance (12.96 Å). Given that Val_84_ displayed the highest displacement from the Leu_249_ residue in the protein crystal structure with the PDB ID: 2YDO, it was selected as the best candidate for shortlisting candidates of A_2A_R agonists.

### Ligand preparation

39 potent agonists and 38 potent antagonists of the A_2A_R (Uniprot ID: P29274) with EC_50_ and IC_50_ values less than 1 µM and confidence scores equal to 9 were manually extracted from ChEMBL. 133 A_2A_R inactives were extracted from PubChem using SQL and the eleven purchasable triazoloquinazolines were selected from the ZINC database. The entire set of ligands were prepared for docking into the orthosteric site of the A_2A_R protein crystal structure, with LigPrep 2.5 [48]. using the default settings and the Epik option, which introduces energy penalties associated with ionization and tautomerization [49].

### Receptor preparation

Docking with Glide [50] was performed against the human A_2A_R protein crystal structure (PDB IDs: 2YDO and 5IU4). The protein structures were prepared using the Protein Preparation Wizard of Maestro 9.3 [51], following the default protocol, which accounts for energy refinement, hydrogen addition, pKa assignment, side-chain rotational isomer refinement, and addition of missing residues and side-chains with Prime 3.1 [52]. Resolved water molecules were discarded, and the structure was centered using the co-crystallized ligand as the center of the receptor grid generated for each protein structure. The co-crystal structures of A_2A_R with Adenosine (PDB ID: 2YDO) and with ZM241385 (PDB ID: 5IU4) were selected as target structures.

### Enrichment of agonists by the A_2A_R docking model (PDB ID: 2YDO)

In an attempt to validate the A_2A_R docking model, the set of prepared A_2A_R agonists, antagonists and inactives were docked using Glide against the prepared protein structure.

The Glide docking parameters used were extra precision (XP) and flexible ligand sampling, which obtained the best separation for the medians of docking score distributions for agonists versus antagonists and agonists versus inactives of the A_2A_R. This implies that this docking model enriches the agonists. Additional file 1: Figure S7 shows the separation of the medians for the A_2A_R docking model: (A) − 11.24 (agonists) (B) − 7.88 (antagonists) and (C) − 6.74 (inactives). Statistical analysis was performed with R using a Mann-Whitney test on the agonist and antagonist docking score distributions, as well as agonist and inactive docking score distributions. The differences in medians were significant at a p value of less than 0.05 [33].

### Cut‐off generation for compound selection as candidates of A_2A_R agonists from the docking model

The Matthews correlation coefficient (MCC), which takes into account true and false positives (agonists) and negatives (antagonists), was computed (using a Python script [33]) for the docking scores of the agonists and antagonists against the A_2A_R docking model. A search was performed for a docking score threshold that gave the highest MCC in order to shortlist promising candidates of A_2A_R agonists, which displayed docking scores that are lower than the score with the highest MCC, and this gave a threshold of -7.33 for the A_2A_R docking model.

### Docking

The eleven purchasable triazoloquinazolines, which were prepared with LigPrep, were docked against the A_2A_R protein crystal structure (PDB ID: 2YDO). The Glide docking parameters used were extra precision (XP) and flexible ligand sampling. The parameters were deduced from docking experiments using known actives and inactives against the protein-docking model. The A_2A_R protein is fairly rigid as assessed by thermal stability (B factor) in Glide [53]. Six triazoloquinazolines (**1**–**6)** displayed docking scores that are lower than − 7.33, which was the docking score with the highest MCC for the known agonists and antagonists. Their chemical structures are depicted in Fig. 1. Additionally compounds **1**, **4** and **5** (with the highest predicted affinities and the most potent agonists identified), compound **6** (which did not exhibit any agonist activity), CHEMBL3799351 (an antagonist with an IC_50_ = 4.35 nM and confidence score equal to 9) and CGS21680 (the selective and potent A_2A_R agonist) and adenosine (a non-selective adenosine receptor agonist), were docked into the inactive form of the A_2A_R protein crystal structure (PDB ID: 5IU4) for MD simulation and analysis. The six triazoloquinazolines (**1**–**6**) were then shortlisted for validation as A_2A_R agonists in relevant biochemical assays.

### MD simulations

Based on a structural analysis of the available A_2A_R crystal structures, the distance between the α-carbons of Val_84_ in TM3 and Leu_249_ in TM6 was selected for investigation as a conformational descriptor for receptor activation. The two A_2A_R co-crystallized structures (PDB IDs: 5IU4 and 2YDO), which exhibited the largest difference in α-carbon distances between Val_84_ in TM3 and Leu_249_ in TM6 (12.96 Å versus 14.53 Å respectively), were selected for molecular dynamics simulation. Subsequently, compounds **1**, **4**, **5**, and **6** that were docked into the orthosteric site of the inactive form of the A_2A_R protein crystal structure (PDB ID: 5IU4) were subjected to a 100 ns MD simulation protocol. Likewise, CHEMBL3799351, CGS21680 and adenosine were docked into the orthosteric site of the inactive form of the A_2A_R protein crystal (PDB ID: 5IU4) to obtain simulations of control compounds. The apo structure (PDB ID: 5UI4) was also selected for the same analysis.

The starting structures were prepared using Maestro 9.3 following the default procedure for protein preparation. The protocol adds missing residues and sidechain information with Prime 3.1 [52], and uses the “Cap termini” option that adds the coordinates to the residue. Next, “Analyze network” in the interactive hydrogen bond optimizer was used to check on the assignments of hydrogen orientations in the hydrogen bonding network. They were subsequently optimized. All MD simulations described in this study were performed using Desmond 3.2, available in the Schrödinger software package Release 2016-3 with the default force field OPLS3 [54]. An orthorhombic box was used to build the model systems with periodic boundary conditions in an isothermal–isobaric ensemble with a constant number of particles (NPT ensemble). The system temperature was kept at 300 K, and the pressure was kept at atmospheric pressure. The definition of transmembrane regions was taken from the OPM database [55]. The receptor structures were embedded in a pre-equilibrated palmitoyloleoyl-phosphatidylcholine membrane (bilayer) and solvated with simple point charge water and 0.15 M NaCl. All other parameters were set to default values (refer to Additional file 1: Table S6 in supporting information). The 100 ns simulations were carried out with Desmond 3.2 *via* command line on the computer cluster CALCULON (University of Cambridge) by using 20 central processing units. For each compound, the simulations were performed twice, and the trajectories obtained were analyzed with the software VMD. Then plots were obtained for the RMSD values of His_250_ in TM6, and the α-carbons distances between Val_84_ in TM3 and Leu_249_ in TM6 for the simulated systems over 100 ns using the seaborn library [56]. The same protocol was repeated for the 500 ns simulations for compounds **1, 5**, CGS21680, and the A_2A_R protein crystal structures (PDB IDs: 2YDO and 5UI4) (each performed in duplicate).

### Materials

Triazoloquinazolines **1**–**6** were supplied from Ambinter (Orléans, France), and CGS21680, NECA and ZM241385 from Tocris Biosciences (Abingdon, UK) (%purity ≥ 95). All compounds were stored in 10 mM stock solutions in DMSO. Rolipram was purchased from Cayman chemicals (Michigan USA), and other laboratory reagents were from Sigma-Aldrich (Poole, UK), of analytical grade.

### Mammalian cell culture

CHO-K1 (gifted by Dr. Ewan St. John Smith, University of Cambridge, UK) CHO-K1-A_2A_R and CHO-K1-A_3_R cells (gifted by Prof. Karl-Norbert Klotz, University of Wuerzburg, Germany), were routinely cultured in Hams F-12 nutrient mix, supplemented with 10 % fetal bovine serum (FBS). H520, H1563, H1792 and LK-2 cells (gifted by Dr. Whalid Khaled, University of Cambridge, UK) were grown in RPMI media + 10 % FBS. All media was further supplemented with 1X antibiotic, antimycotic solution (Sigma Aldrich, Poole, UK). Culturing of all cell types was done at 37 °C in a humidified atmosphere containing 5 % CO_2_.

### Generation of CHO-K1 cell line stably expressing the A_2A_R

CHO-K1 cells stably expressing the A_2A_R cells were generated *via* transfection with 500 ng pcDNA3.1-A_2A_R (cDNA.org), per well of a 24-well plate, which was performed with FuGENE HD (Promega, Wisconsin, USA), at a 1:3 (w/v) DNA:FuGENE ratio. Prior to adding 800 µg/ml G418 (Sigma Aldrich, Poole, UK), the cells were further cultured for 48 hours. Then every 48 hours, G418 containing media were replaced until foci of cells were attained, which were left to grow to 100 % confluency. Afterwards, each well was tested for the ability of CGS21680 to elevate cAMP, performing further culturing with appropriately responding clones as described.

### Phosphodiesterase 10A inhibition assays

A PDE10A assay kit (BPS Bioscience, San Diego, CA) was used to test the PDE10A inhibition of compounds **1**–**6** as described in the manufactures protocol. 400 pg of purified PDE10A was used per reaction, and the plates were read using a TECAN infinite M200.

### Yeast methods

Generation of yeast strains was done according to previously reported protocols, and they have been routinely grown as previously described [45]. Yeast cells expressing either the A_1_R, A_2A_R, or A_2B_R were treated with either NECA, CGS21680 or compounds **1**–**6**, in order to measure the activity of each, as previously described [45].

### Bioluminescence Resonance Energy transfer (BRET)-based ligand binding of triazoloquinazolines

HEK293T cells were seeded in 6-well plates at density of 10^6^ cells/well and grown overnight at 37^o^C in DMEM/F12 medium supplemented with 10 % FBS and 1 % antibiotic/antimycotic. Cells were then transfected with 1.5 µg Nluc-A_2A_R construct (a gift from Dr. Stephen Briddon, and Professor Steven Hill, University of Nottingham, UK) per well using PEI method. The ratio of DNA:PEI used for this transfection was 1:6 in 150 mM NaCl [57]. Cells were grown overnight, harvested and seeded at a density of 50,000 cells/well into PLL-coated white 96-well plates (Greiner, UK) in complete growth medium and cultured for a further 24 h. On the day of the assay, culture medium was discarded and replaced by 80 µl BRET buffer which consist of PBS supplemented with 0.9 mM CaCl_2_, 0.5 mM MgCl_2_, and 1 % BSA (w/v). The assay was started by adding 10 µl of furimazine, the substrate of Nluc (Promega, UK) (diluted in BRET buffer) to a final concentration of 0.4 µM and the plate was incubated in the dark at room temperature for 5 minutes.

For association-dissociation kinetic experiments, following furimazine incubation, 40 nM of CA200645 (purchased from Hello Bio, Bristol, UK) was added and the plate was immediately read. After 19 minutes stimulation, CGS21680 was injected to give a final concentration of 10 µM. Whereas for competition association assays, after incubation with furimazine, CA200645 (300 nM) in the presence of unlabelled ligand (in a range of 10 pM to 100 µM) were added simultaneously. BRET signal was recorded for either 50 minutes or 20 minutes, for kinetic experiments or competition assay as appropriate, on a Mithras LB940 plate reader allowing sequential integration of signal detected from fluorescent probe CA200645 and Nluc. The BRET ratio corresponds to the ratio of light emission from acceptor (red fluorescent probe, long pass filter > 610 nm) over donor (Nluc 460 nm). Ligand-induced ΔBRET was used to construct the association-dissociation kinetic of the fluorescence probe and competition binding curve of unlabelled ligands.

To determine K_D_ value of CA200645, the signals from kinetic assay was fit into “association then dissociation” equation which was built in Prism 8.4. With the purpose of validating BRET-based competition assay, several reference compounds including CGS21680, NECA, and isoprenaline were also included. Binding affinities were calculated from competition assay by fitting data to non-linear regression using “one-site, fit Ki” model built in Prism 8.4. The concentration and K_D_ values of ‘hot’ ligand were set to 300 nM and 65 nM, respectively.

### cAMP accumulation assays

Prior to assay, harvesting of cells was performed with trypsin containing 0.05 % EDTA, they were then washed with PBS, and subsequently resuspended in stimulation buffer (PBS Proliferation assays containing 0.1 % BSA and 25 µM rolipram). Seeding of cells was done at 2000 cells well^− 1^ of a 384-well white optiplate, and then they were stimulated at room temperature with compounds **1**–**6** (ranging 100 pM-10 mM) for 30 minutes. The cells were subsequently lysed, and the measurement of cAMP levels was done using a LANCE cAMP detection kit (PerkinElmer), and the plates were read with a Mithras LB940 microplate reader.

### Proliferation assays

To test the effect of compounds **1**–**6** upon proliferation, various cell types were seeded onto clear 96-well plates at proper densities for each; CHO-K1 (2000 cells well^− 1^), CHO-K1-A_2A_R (2000 cells well^− 1^), H520 (2500 cells well^− 1^), H1563 (2500 cells well^− 1^), H1792 (2500 cells well^− 1^), LK-2 (2500 cells well^− 1^). This was done in suitable media, and they were cultured for 24 hours. After the subsequent addition of compounds **1**–**6** (ranging 316 nM − 100 µM), cells were allowed to grow further for 72 hours. Quantification of changes in cell number was done by adding 5 µl CCK-8 reagent to each well, accompanied by incubation at 37 °C for 1–3 hours. The determination of OD_450_ was done using a Mithras LB940 micro-plate reader at 450 nm.

### RT-PCR

Extraction of RNA from H520, H1792, H1563 and LK-2 cells was done using a RNAqueous®-4PCR Total RNA Isolation Kit (Life Technologies, Paisley, UK) as per the manufacturer’s instructions. Then, DNAse I treatment was performed to remove the contamination by genomic DNA. Subsequently, the quantification of the degree of purity of RNA samples was performed using a NanoDrop™ Lite spectrophotometer (Thermo Scientific, UK). The samples that were used in cDNA synthesis are those of yields > 100 ng/µL and A_260_/_280_ ratios > 1.9. The cDNA synthesis was done using a QuantiTect reverse transcription kit (Qiagen, Manchester, UK), for which a total of 1 µg of freshly isolated RNA was consumed per reaction. RT-PCR was subsequently implemented according to what has been previously reported[58]. The RT-PCR that has been done used gene specific primers to human: *GAPDH* (Sense 5’–TGCACCACCAACTGCTTAGC– 3’; Antisense 5’-GGCATGGACTGTGGTCATGAG–3’), *A*_*1*_*R* (Sense 5’-CCACAGACCTACTTCCACACC–3’; Antisense 5’–TACCGGAGAGGGATCTTGACC–3’, Primerbank ID − 115305570C1), *A*_*2A*_*R* (Sense 5’-CGCTCCGGTACAATGGCTT–3’; Antisense 5’–TTGTTCCAACCTAGCATGGGA–3’, Primerbank ID − 156142194C1), *A*_*2B*_*R* (Sense 5’–TGCACTGACTTCTACGGCTG–3’; Antisense 5’–GGTCCCCGTGACCAAACTT–3’, Primerbank ID − 22907046C1), *A*_*3*_*R* (Sense 5’–GGCCAATGTTACCTACATCACC–3’; Antisense 5’–CCAGGGCTAGAGAGACAATGAA–3’, Primerbank ID − 4501953A1) and *PDE10A* (Sense 5’-TGA TGACTTTTCTCTCGACGTTG–3’; Antisense 5’–AAGCCACCTACACAGTGTCTC–3’, Primerbank ID − 359465520C1). Then, gel electrophoresis (using 2 % agaorse gels) was performed to resolve PCR products. The imaging of gels was subsequently done using a G Box iChemi gel documentation system employing GeneTools analysis software (Syngene, Cambridge, UK) and densitometry.

### Data analysis

Data analysis was performed using GraphPad Prism 8.2.1 (San Diegeo, CA). All data for *β*-galactosidase assays were normalized to the responses resulting from NECA stimulation, whereas the data for cAMP inhibition/accumulation assays were normalised to those obtained upon stimulation with 100 µM Forskolin or CGS21680. As for proliferation assays, the normalization of all data was done relative to the responses obtained upon treating cells with 1 % (v/v) DMSO. Subsequently, a three-parameter logistic equation was used for fitting each set of normalized data *β*-galactosidase or cAMP data, in order to calculate pEC_50_/pIC_50_ and E_max_ values. Also, the fitting of the proliferation data was done using a three-parameter logistic equation constraining the basal value to 100 and the system maximum to the I_Max_ value obtained for compound **2**, since it elicited the maximum inhibition of cellular proliferation in all cell types tested. A one-way ANOVA with Dunnett’s post-test, or Student’s t-test was used to assess the statistical significance for all assays, where p < 0.05 was considered to be significant.

## Supplementary Information

**Additional file 1.** Discussion. Additional figures and tables
