# Supplementary material for: Structure‐based identification of dual ligands at the A2AR and PDE10A with anti‐proliferative effects in lung cancer cell‐lines
Source: J Cheminform. 2021 Mar 3;13:17. doi: 10.1186/s13321-021-00492-5 (PMC7927403; doi:10.1186/s13321-021-00492-5)
Supplement: Supplementary file 1 — Additional file 1. Discussion. Additional figures and tables [file 13321_2021_492_MOESM1_ESM.docx]

**Supporting Information**

Structure-based identification of dual ligands at A_2A_R and PDE10A with anti-proliferative effects in lung cancer cell-lines

Leen Kalash^1,6, ‡^, Ian Winfield^1,2, ‡^, Dewi Safitri^2,3‡^, Marcel Bermudez^1,4^, Sabrina Carvalho^2^, Robert Glen^1,5^, Graham Ladds^2,^ *, Andreas Bender^1,^ *

1. Centre for Molecular Informatics, Department of Chemistry, University of Cambridge, Lensfield Road, Cambridge, CB21EW, United Kingdom

2. Department of Pharmacology, University of Cambridge, Tennis Court Road, Cambridge, CB2 1PD, United Kingdom

3. Pharmacology and Clinical Pharmacy Research Group, School of Pharmacy, Bandung Institute of Technology, Bandung 40534, Indonesia

4. Institute of Pharmacy, Freie Universität Berlin, Königin-Luise-Straße 2 und 4, 14195 Berlin, Germany

5. Department of Metabolism Digestion and Reproduction, Faculty of Medicine, Imperial College London, London, SW7 2AZ, United Kingdom

6. Present Address: GlaxoSmithKline, Gunnels Wood Road, Stevenage, Hertfordshire, SG1 2NY, United Kingdom.

* Dr Graham Ladds, Department of Pharmacology, University of Cambridge, Tennis Court Road, Cambridge, CB2 1PD. Tel: +44 (0) 1223 334020. <https://orcid.org/0000-0001-7320-9612>. E-mail: grl30@cam.ac.uk.

*Dr Andreas Bender, Department of Chemistry, University of Cambridge, Lensfield Road, Cambridge, CB2 1EW. Tel: +44 (0) 1223 762983. <https://orcid.org/0000-0002-6683-7546>. E-mail: ab454@cam.ac.uk

‡These authors contributed equally.

**Table of Contents**

1. Discussion ……………………………………………………………………………………............ S2-S3

MD simulation analysis of the His250 residue ……………………………………………………… S2-S3

1. Figures ……………………………………………………………………………………………….. S4-S11

Figure S1. The moving average trend-lines (bin-size of 10 frames) of RMSD values of His_250_ …… S4

Figure S2. The RMSD distributions for the His_250_ residue ………………………………………….. S5

Figure S3. Comparison of moving average trend-lines (bin-size of 10 frames)

of RMSD values of His_250_ to Leu_49_……………………………… …………………….. S6

Figure S4. Moving average trend-lines (bin-size of 20 frames) for the Val_84_-Leu_24_ Cα distances …. S7

Figure S5. RT-PCR analysis of CHO-K1 cells …………………………………………………….. S8

Figure S6. Anti-proliferative effects in CHO-K1-A_2A_R cells ……………………………………… S9

Figure S7. The docking scores distributions of A_2A_R ligands ……………………………………… S11

1. Tables …………………………………………………………………………………….……… S12-S16

Table S1. Table of the inter-residue distance values for Val_84_-Leu_249_ …………………………….. S12

Table S2. pIC_50_ and I_max_ of the responses for the anti-proliferative effects in

CHO-K1 and CHO-K1-A_2A_R cells …………………………………………………..…. S13

Table S3. pEC_50_ and E_max_ values for cAMP accumulation in lung carcinoma cell-lines …………. S14

Table S4. Anti-proliferative effects in lung carcinoma cell-lines ………………………………… S15

Table S5. Simulations parameters …………………………………………………………………. S16

**I. Discussion**

### *MD simulation suggests that the conformational change of the His_250_ residue contributes to shaping the orthosteric site pocket to favor selectivity for A_2A_R agonists*

To investigate further whether a conformational change of His_250_ contributes to shaping the orthosteric site to favour selectivity for agonists at A_2A_R, an RMSD analysis was performed (100ns molecular dynamics). This is an approach commonly used to determine conformational flexibility upon ligand binding. The analysis was performed for compounds **1** and CGS21680 (selective and potent A_2A_R agonists), compound **4** and Adenosine (non-selective adenosine receptor agonists) docked to the A_2A_R structure (PDB ID: 5IU4). The RMSD values of His_250_ over the period of the simulation are represented as the moving average trend-lines (bin-size of 10 frames) in Figure S1.

A similar trend is seen for compounds **1** and CGS21680, where the RMSDs of His_250_ increase over the simulation time, converging towards similar values. It appears that that a conformational change is occurring in this residue upon the binding of a selective A_2A_R agonist. The greater increase in RMSD values observed for compound **1** in comparison to CGS21680 could be related to the higher degree of selectivity that this compound exhibits (as an A_2A_R agonist) over the other adenosine receptor subtypes. This contrasts with CGS21680 that additionally exhibits agonist activity at the A_3_R receptor subtype. In contrast, for compounds **4** and Adenosine (non-selective agonists) the RMSD values are relatively similar over the 100 ns simulation. The RMSD distributions for the His_250_ residue in the last 50 ns of the MD simulation performed for compounds **1**, **4**, CGS21680, and Adenosine docked to the A_2A_R, are plotted in Figure S2. The separation in RMSD distributions for the selective A_2A_R agonists (**1** and CGS21680) versus the non-selective A_2A_R agonists (**4** and Adenosine) is clearly illustrated. A Kolmogorov-Smirnov test, which included statistical analysis of the pair-wise RMSD distributions of His_250_ in selective versus non-selective agonist bound A_2A_R structures was performed. This yielded a p value < 2.2. x 10^-16^, indicating that the difference between the two types of distributions is statistically significant. Furthermore, the RMSD values of His_250_ for the A_2A_R structures bound to compound **1** and CGS21680 were compared to a reference residue (Leu_249_), over the 100 ns MD simulations, as illustrated in Figure S3. It is evident that the RMSD values of Leu_249_ are relatively constant for both A_2A_R structures bound to compound **1** and CGS21680. This emphasizes that the increase in RMSD values for His_250_ is characteristic of its conformational change upon selective A_2A_R agonist binding. Hence, the comparison in RMSD values for the His_250_ residue in A_2A_R structures bound to selective versus non-selective A_2A_R agonists suggests that the conformational change of this residue contributes to shaping the orthosteric site pocket to favour the selectivity of A_2A_R agonists.

**II. Figures**

**
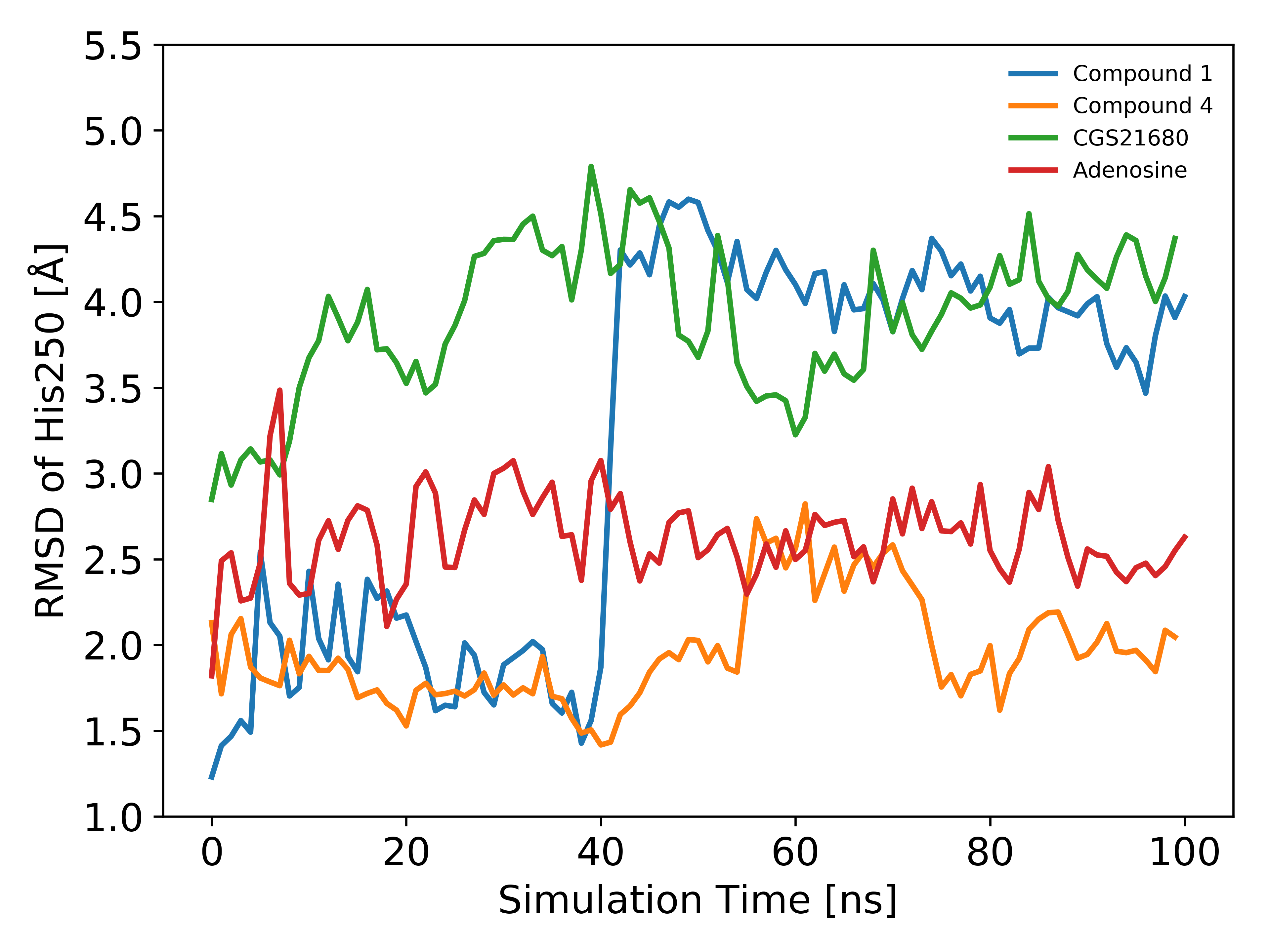
**

**Figure S1**. The moving average trend-lines (bin-size of 10 frames) of RMSD values of His_250_ for compounds **1**, **4**, and CGS 21680 (the selective and potent A_2A_R agonist) and Adenosine (the non-selective adenosine receptor agonist), docked to the inactive form of the A_2A_R protein crystal structure (PDB ID: 5IU4) over a 100 ns simulation. The RMSD values of His_250_ for the docked structures of compounds **1** and CGS 21680 (the two selective A_2A_R agonists) behave similarly by increasing and converging towards similar RMSD values. This indicates that a conformational change occurs in this residue upon A_2A_R agonist binding. In contrast, the RMSD values in the case of the docked compounds **4** and Adenosine (the non-selective adenosine receptor agonists) are relatively constant. Hence, this suggests that the conformational change of this residue contributes to shaping the orthosteric site pocket to favour the selectivity of A_2A_R agonists.


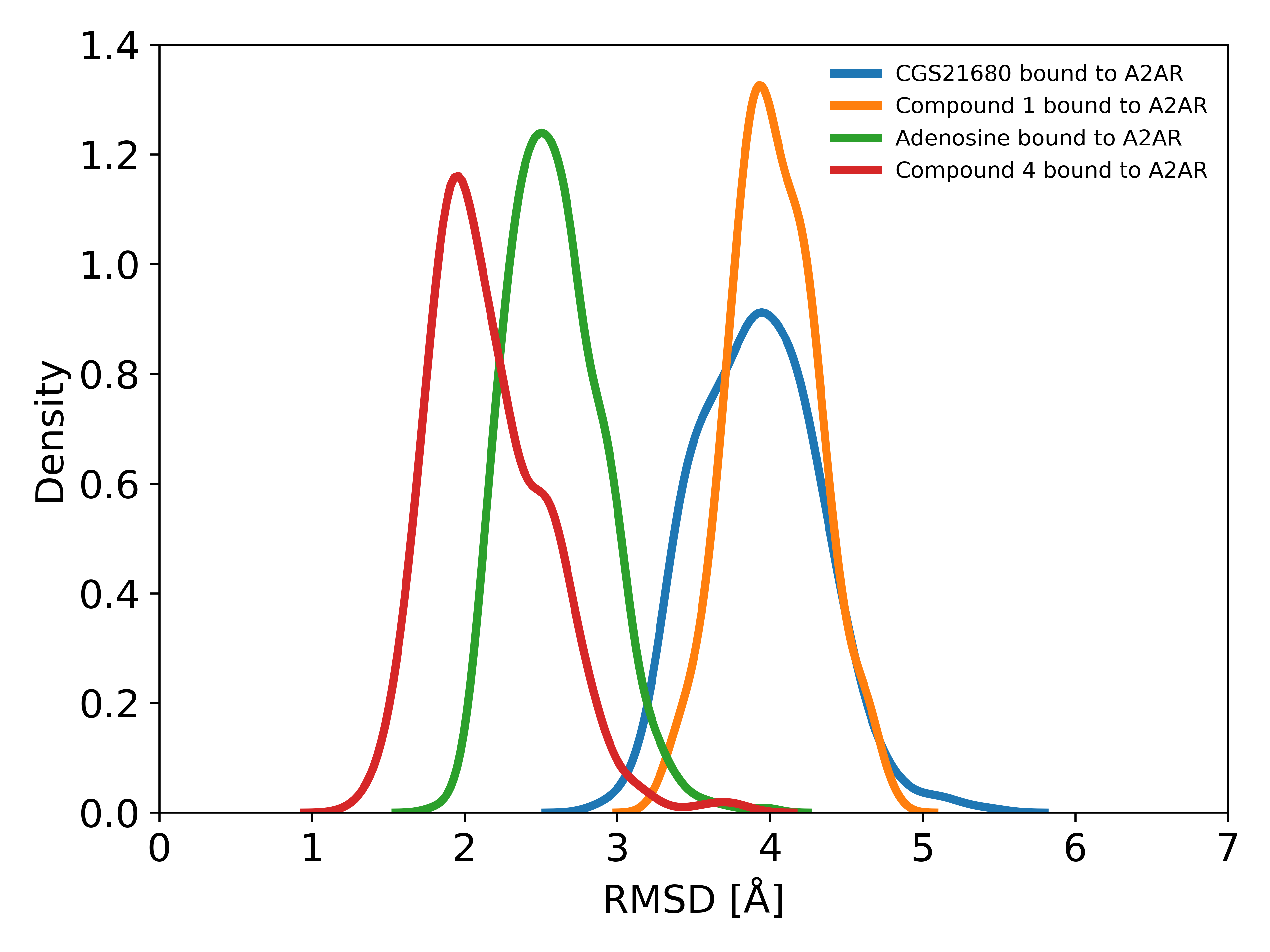


**Figure S2**. The RMSD distributions for the His_250_ residue in the last 50 ns of the MD simulation performed for compounds **1**, **4**, CGS 21680, and Adenosine, docked to the A_2A_R. The separation in RMSD value distributions for the selective A_2A_R agonists (**1** and CGS 21680) versus the non-selective A_2A_R agonists (**4** and Adenosine) is statistically significant by Kolmogorov-Smirnov test, for the pair-wise RMSD distributions (selective versus non-selective agonist bound A_2A_R structures) with a p value less than 2.2. x 10^-16^.

###
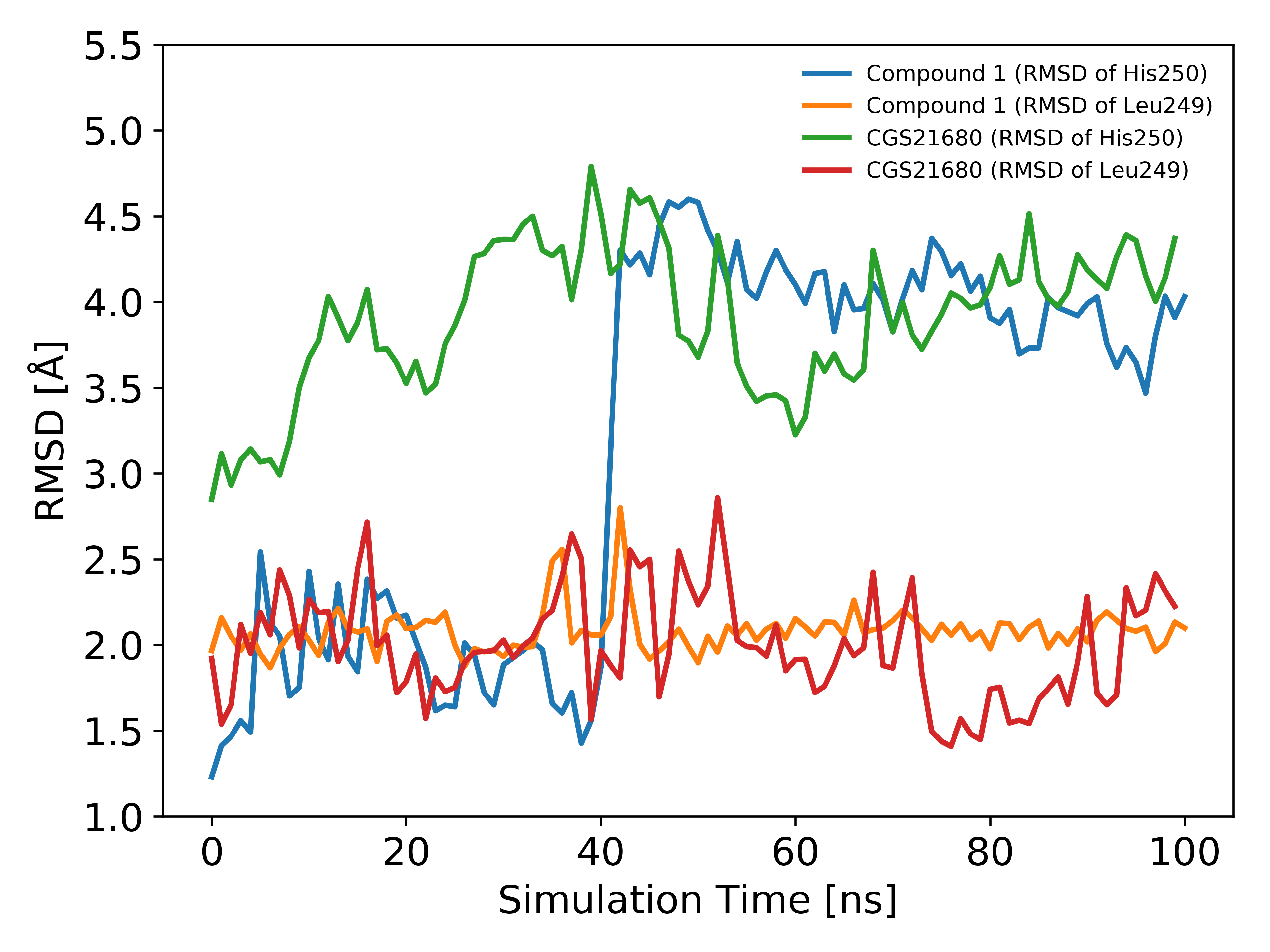


**Figure S3**. The moving average trend-lines (bin-size of 10 frames) of RMSD values of His_250_ for the A_2A_R structures bound to compound **1** and CGS 21680 were compared to those of a reference residue (Leu_249_) over a 100 ns MD simulation. The RMSD values of Leu_249_ are relatively constant for both A_2A_R structures bound to compound **1** and CGS 21680 emphasizing that increase in RMSD values for His_250_ is characteristic of its conformational change upon selective A_2A_R agonist binding.

**
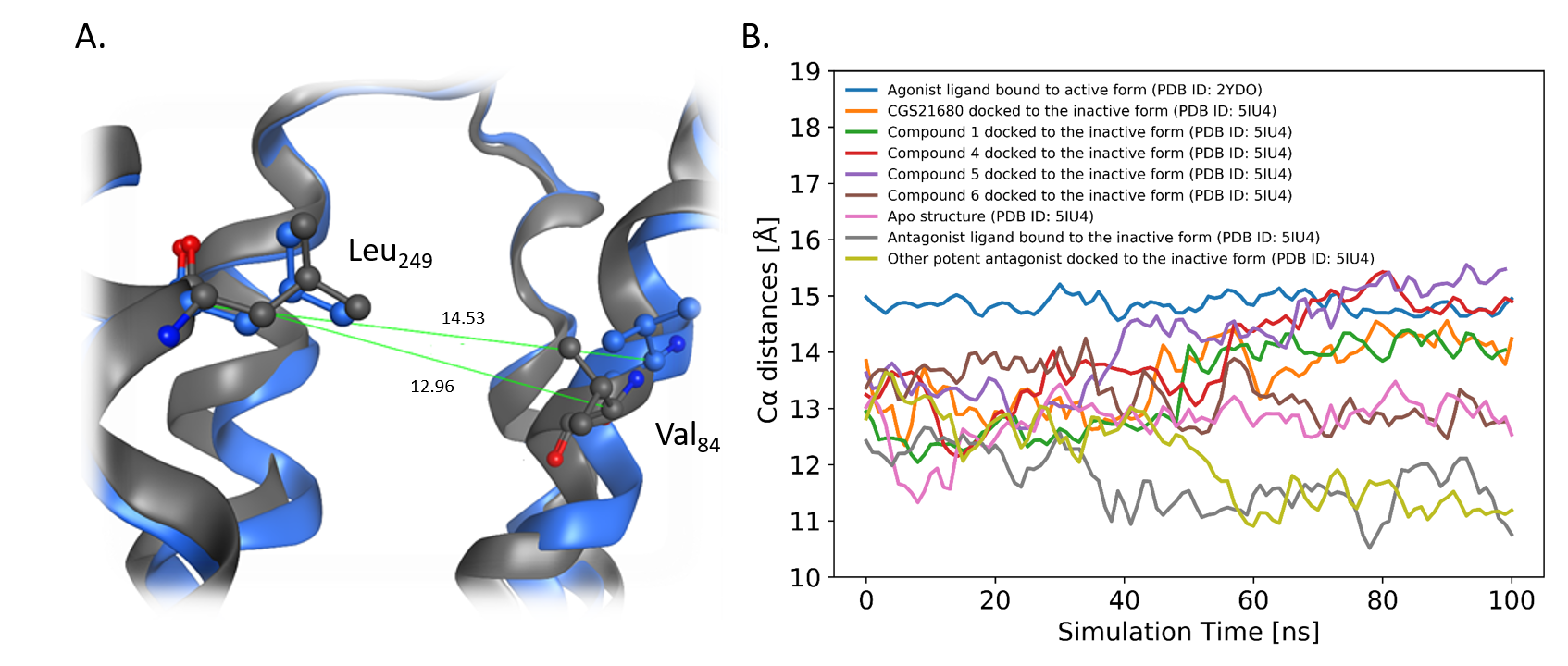
**

**Figure S4**. **A.** The aligned and superimposed active (PDB ID: 2YDO in blue) and inactive forms (PDB ID: 5IU4 in grey) of the A_2A_R protein crystal structures. The Val_84_-Leu_249_ Cα distances were measured for the active and inactive forms and were 14.53 and 12.96Å respectively **B.** The moving average trend-lines (bin-size of 20 frames) are for the Val_84_-Leu_249_ Cα distances of the apo-structure (PDB ID: 5IU4) and the docked and the co-crystallized structures (PDB ID: 5IU4 and 2YDO same color code of 3A) of the A_2A_R over a 100 ns simulation. Compounds **1**, **4**, **5**, and **6**, a potent antagonist (CHEMBL3799351), and the selective and potent A_2A_R agonist (CGS21680) are docked into the inactive form of the A_2A_R protein crystal structure (PDB ID: 5IU4). The variation in computed distances for compounds **1**, **4**, **5** and CGS21680 were similar, where all increased in their average distances time moving towards the average distance observed in the dynamics of the active protein crystal structure (PDB ID: 2YDO). The average distances for compound **6**, and the apo-structure were similar, which explains why compound **6** did not exhibit any agonist activity. Hence, the increase in the Val_84_-Leu_249_ inter-residue distance upon A_2A_R agonist binding serves as a good conformational descriptor for receptor activation by the A_2A_R ligands.

**Figure S5**. Products obtained from RT-PCR using gene specific primers for GAPDH and PDE10A on cDNA. These were produced from RNA extracted from CHO-K1 cells, then shown in representative (n = 3) agarose gel. The RT-PCR analysis reveals that CHO-K1 cells endogenously express PDE10A.

**Figure S6.** Stimulation by Forskolin and compounds **1**, and **3**-**5** showed anti-proliferative effects in CHO-K1 cells, which was enhanced when the A_2A_R is stably expressed in CHO-K1-A_2A_R cells. As for compound **2**, it displayed anti-proliferative effects in both cell types, which suggests that it might be toxic. CHO-K1 or CHO-K1-A_2A_R cells were seeded into 96 well plates and cultured for 24 hours before being stimulated with compounds for 72 hours: Forskolin (*n* = 6) (A), CGS 21680 (*n* = 4) (B), compound **1** (*n* = 6) (C), compound **2** (*n* = 6) (D), compound **3** (*n* = 6) (E), compound **4** (*n* = 6) (F), compound **5** (*n* = 6) (G) or compound **6** (*n* = 6) (H), where CCK-8 was used to determine cell number. Data is represented relative to the cell number obtained upon treatment with 1% DMSO, ± SEM


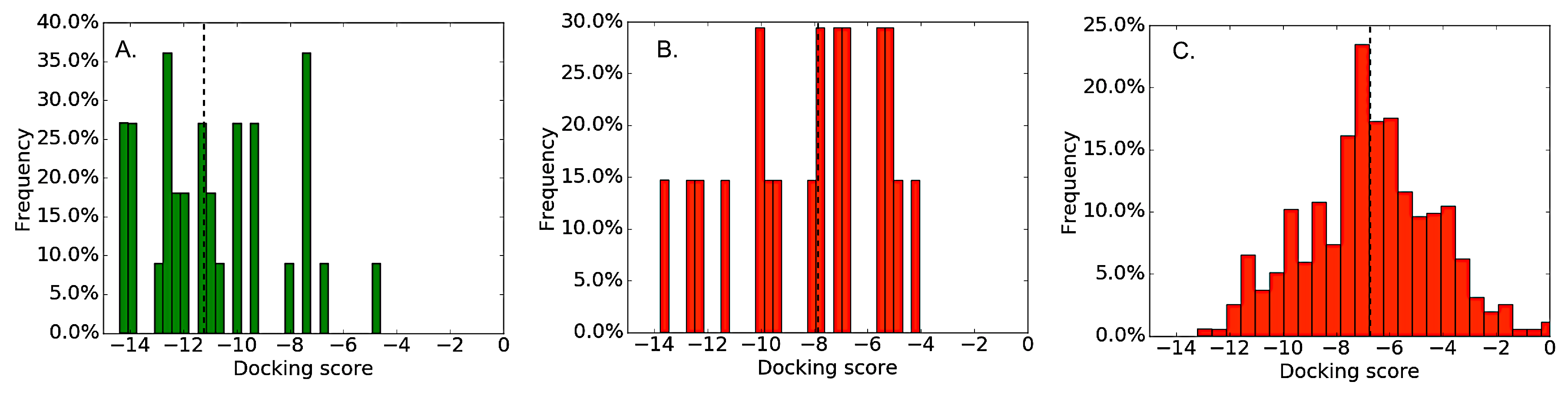


**Figure S7.** A good separation was obtained for the medians (dashed lines) of the docking score distributions for **A.** agonists (-11.24) **B**. versus antagonists (-7.88) and **C.** versus inactives (-6.74) confirming that the agonists are enriched. Statistical analysis was performed with R using a Mann-Whitney test^1^ on the agonist and antagonist docking score distributions, as well as agonist and inactive docking score distributions. The differences in medians were significant for a p value less than 0.05.

**II. Tables**

**Table S1.** The inter-residue distance values for Val_84_-Leu_249_ for all the active and inactive forms of the A_2A_R protein crystal structures

| Active forms of the A_2A_R | | Inactive forms of the A_2A_R | |
| --- | --- | --- | --- |
| PDB ID | Val_84_-Leu_249_  Distance (Å) | PDB ID | Val_84_-Leu_249_  Distance (Å) |
| 4UG2 | 14.30 | 5IU4 | 12.96 |
| 4UHR | 14.38 | 3UZA | 13.15 |
| 3QAK | 14.46 | 5K2A | 13.01 |
| 2YDO | 14.53 | 4EIY | 13.07 |
| 2YDV | 14.35 | 3EML | 13.36 |
|  | | 5NM2 | 13.06 |
|  |  | 5JTB | 12.98 |
|  |  | 5UVI | 13.07 |
|  |  | 5UIG | 12.98 |

**Table S2.** Potency (pIC_50_) and I_max_ of the responses for the anti-proliferative effects obtained upon simulating by Forskolin, CGS 21680 and triazoloquinazolines **1**-**6** in CHO-K1-A_2A_R and CHO-K1 cells

|  | **CHO-K1-A_2A_R** | | | **CHO-K1** | | | **CHO-K1-A_2A_R vs CHO-K1** | |
| --- | --- | --- | --- | --- | --- | --- | --- | --- |
|  | **pIC_50_^a^** | **I_max_^b^** | **n** | **pIC_50_^a^** | **I_max_^b^** | **n** | **Δ pIC_50_^c^** | **Δ I_max_^d^** |
| **Forskolin** | 4.43±0.06^***^ | 58.96±2.2^***^ | 6 | 4.46±0.08 | 62.57±3.4 | 6 | -0.03±0.1 | -3.61±4.1 |
| **CGS 21680** | NR | NR^***^ | 4 | NR | NR | 4 | - | - |
| **Cmpd 1** | 4.41±0.07^***^ | 56.34±5.3^***^ | 6 | 3.27±0.17 | 11.05±1.6 | 6 | 1.14±0.24 | 45.29±5.1 |
| **Cmpd 2** | 4.71±0.1^***^ | 74.82±2.4^***^ | 6 | 4.78±0.09 | 78.54±1.4 | 6 | -0.07±0.1 | -3.73±2.8 |
| **Cmpd 3** | 4.82±0.06^***^ | 70.94±1.8^***^ | 6 | 4.13±0.07 | 38.01±1.0 | 6 | 0.69±0.1 | 32.85±2.1 |
| **Cmpd 4** | 4.41±0.1^***^ | 51.90±0.6^***^ | 6 | 3.47±0.20 | 19.51±3.7 | 6 | 0.94±0.2 | 32.39±2.1 |
| **Cmpd 5** | 4.74±0.05^***^ | 73.19±2.1^***^ | 6 | 3.65±0.08 | 20.71±1.4 | 6 | 1.09±0.1 | 52.48±2.6 |
| **Cmpd 6** | 3.52±0.08 | 18.80±0.7^**^ | 6 | 3.37±0.13 | 15.70±1.9 | 6 | 0.15±0.2 | 3.10±2.6 |

Data ± SEM of *n* individual replicates

^a^ Negative logarithm of agonist concentration producing half-maximal inhibition

^b^ Maximal level of inhibition obtained when cells were stimulated with 10 μM agonist relative to that obtained with 1% DMSO treatment

^c^ Change in pIC_50_ between CHO-K1 and CHO-K1-A_2A_R cells (ΔpIC_50_ = pIC_50_(CHO-K1-A_2A_R) - pIC_50_(CHO-K1))

^d^ Change in I_max_ between CHO-K1 and CHO-K1-A_2A_R cells (ΔI_max_ = I_max_ (CHO-K1-A_2A_R) - I_max_ (CHO-K1))

NR – No response observed

Statistical difference, between CHO-K1-A_2A_R cells and CHO-K1 cells, was calculated using pair-wise t-tests, for each agonist (*, p < 0.05, **, p < 0.01, ***, p < 0.001)

**Table S3.** Potency (pEC_50_) and E_max_ values for cAMP accumulation of CGS 21680 and triazoloquinazolines **1**, **3**-**6** stimulated by LK-2, H520, H1792 and H1563 lung carcinoma cell-lines.

|  | **LK-2** | | **H520** | | **H1792** | | **H1563** | |
| --- | --- | --- | --- | --- | --- | --- | --- | --- |
|  | **pEC_50_^a^** | **E_max_^b^** | **pEC_50_^a^** | **E_max_^b^** | **pEC_50_^a^** | **E_max_^b^** | **pEC_50_^a^** | **E_max_^b^** |
| **CGS 21680** | 4.84±0.4 | 7.64±1.6 | 5.48±0.2^**^ | 20.80±1.0 | 5.52±0.1^***^ | 27.29±2.1 | 5.34±0.1^***^ | 15.59±1.6^***^ |
| **Cmpd 1** | N/A | N/A | 5.49±0.2^**^ | 26.86±3.1 | 6.05±0.1^***^ | 32.21±2.8 | 6.42±0.1^***^ | 29.31±1.2^***^ |
| **Cmpd 3** | 5.96±0.2 | 14.12±1.2 | 5.89±0.2^**^ | 22.36±1.5 | 6.39±0.1^***^ | 24.14±1.8 | 6.50±0.2^***^ | 12.46±0.7^***^ |
| **Cmpd 4** | 4.74±0.6 | 15.56±5.0 | 6.36±0.3^**^ | 20.51±2.1 | 7.47±0.1^***^ | 20.46±1.8 | 6.86±0.1^***^ | 10.71±0.8^***^ |
| **Cmpd 5** | N/A | N/A | 5.46±0.2^**^ | 16.28±1.4 | 5.60±0.2^***^ | 27.07±3.4 | 6.82±0.2^***^ | 14.90±0.8^***^ |
| **Cmpd 6** | N/A | N/A | 4.64±0.7^**^ | 12.21±4.9 | 4.72±0.1^***^ | 24.65±2.5 | 5.22±0.2^**^ | 15.11±1.2^***^ |

Data ± SEM of 4-8 individual replicates

^a^ Negative logarithm of agonist concentration producing half-maximal response

^b^ Maximal response observed upon agonist stimulation, as a percentage of that observed upon stimulation with 100 μM forskolin

Statistical difference between each agonist and CGS 21680 was calculated using a one-way ANOVA with Dunnett’s post-test (*, p < 0.05, **, p < 0.01, ***, p < 0.001)

N/R., no response was detected.

**Table S4.** Anti-proliferative effects of CGS 21680, Forskolin and trizaoloquinazolines **1**, **3**-**6** stimulated in LK-2, H520, H1792 and H1563 lung carcinoma cell-lines

|  | **LK-2** | | **H520** | | **H1792** | | **H1563** | |
| --- | --- | --- | --- | --- | --- | --- | --- | --- |
|  | **pIC_50_^a^** | **I_max_^b^** | **pIC_50_^a^** | **I_max_^b^** | **pIC_50_^a^** | **I_max_^b^** | **pIC_50_^a^** | **I_max_^b^** |
| **CGS 21680** | NR | -3.55±2.6 | NR | 0.55±2.0^***^ | NR | -4.07±1.6^***^ | 3.67±0.08 | 18.87±1.8^***^ |
| **Foskolin** | 3.49±0.06 | -19.7±2.0 | 4.07±0.09 | 31.2±2.1^***^ | 3.73±0.05 | 25.9±1.0^***^ | 3.83±0.05 | 29.9±1.1^***^ |
| **Cmpd 1** | NR | -3.58±0.7 | 3.34±0.10 | 16.0±3.8^***^ | 4.00±0.07 | 36.0±2.7^***^ | 4.37±0.04 | 55.3±1.5^***^ |
| **Cmpd 3** | 3.42±0.06 | 12.6±1.8 | 4.17±0.08 | 40.2±2.9^***^ | 3.92±0.05 | 29.4±2.8^***^ | 4.23±0.07 | 49.3±3.7^***^ |
| **Cmpd 4** | NR | -5.23±1.9 | 3.76±0.07 | 25.0±1.5^***^ | 4.51±0.06 | 56.0±0.9^***^ | 4.00±0.07 | 29.9±2.9^***^ |
| **Cmpd 5** | NR | -7.32±1.9 | 3.24±0.30 | 17.9±1.0^***^ | 3.75±0.06 | 31.4±2.0^***^ | 4.34±0.04 | 51.0±0.5^***^ |
| **Cmpd 6** | NR | -15.16±2.1 | NR | -18.2±3.1^***^ | NR | -19.8±5.8^***^ | NR | -7.8±2.1^***^ |

Data ± SEM of 4-8 individual replicates

^a^ Negative logarithm of agonist concentration producing half-maximal inhibition

^b^ Maximal level of inhibition obtained when cells were stimulated with 10 μM agonist relative to that obtained with 1% DMSO treatment

Statistical difference for each agonist compared to its effect upon LK-2 cells was calculated using a one-way ANOVA with Dunnett’s post-test (*, p < 0.05, **, p < 0.01, ***, p < 0.001)

**Table S5.** Default values of MD simulations parameters

| Parameters | Default settings |
| --- | --- |
| Time step | 2 fs |
| Thermostat method | Nose-Hoover chain |
| Barostat method | Martyna-Tobias-Klein |
| Solvent model | Predefined SPC |
| Ion placement | Neutralize by adding Na^+^ ions then recalculate |
